# Diacylglycerols and Lysophosphatidic Acid, Enriched on Lipoprotein(a), Contribute to Monocyte Inflammation

**DOI:** 10.1161/ATVBAHA.123.319937

**Published:** 2024-01-25

**Authors:** Kim E. Dzobo, Arjen J. Cupido, Barend M. Mol, Lotte C.A. Stiekema, Miranda Versloot, Maaike Winkelmeijer, Jorge Peter, Anne-Marije Pennekamp, Stefan R. Havik, Frédéric M. Vaz, Michel van Weeghel, Koen H.M. Prange, Johannes H.M. Levels, Menno P.J. de Winther, Sotirios Tsimikas, Albert K. Groen, Erik S.G. Stroes, Dominique P.V. de Kleijn, Jeffrey Kroon

**Affiliations:** Departments of Experimental Vascular Medicine (K.E.D., M.V., M.W., J.P., A.-M.P., S.R.H., J.H.M.L., A.K.G., J.K.), Amsterdam University Medical Center (UMC), University of Amsterdam, Amsterdam Cardiovascular Sciences, the Netherlands.; Vascular Medicine (A.J.C., L.C.A.S., E.S.G.S.), Amsterdam University Medical Center (UMC), University of Amsterdam, Amsterdam Cardiovascular Sciences, the Netherlands.; Amsterdam Cardiovascular Sciences, Atherosclerosis and Ischemic Syndromes, the Netherlands (K.E.D., M.V., J.K.).; Department of Vascular Surgery, University Medical Centre Utrecht, the Netherlands (B.M.M., D.P.V.d.K.).; Core Facility Metabolomics (F.M.V., M.v.W.), Amsterdam UMC, University of Amsterdam, the Netherlands.; Department of Medical Biochemistry, Amsterdam Infection and Immunity (K.H.M.P., M.P.J.d.W.), Amsterdam UMC, University of Amsterdam, the Netherlands.; Division of Cardiovascular Medicine, Sulpizio Cardiovascular Center, University of California San Diego, La Jolla (S.T.).; Laboratory of Angiogenesis and Vascular Metabolism, Flanders Institute for Biotechnology (VIB)-KU Leuven Center for Cancer Biology, VIB, Belgium (J.K.).; Laboratory of Angiogenesis and Vascular Metabolism, Department of Oncology, KU Leuven and Leuven Cancer Institute, Belgium (J.K.).

**Keywords:** diglycerides, inflammation, lipidomics, lipoprotein(a), monocytes

## Abstract

**BACKGROUND:**

Oxidized phospholipids play a key role in the atherogenic potential of lipoprotein(a) (Lp[a]); however, Lp(a) is a complex particle that warrants research into additional proinflammatory mediators. We hypothesized that additional Lp(a)-associated lipids contribute to the atherogenicity of Lp(a).

**METHODS:**

Untargeted lipidomics was performed on plasma and isolated lipoprotein fractions. The atherogenicity of the observed Lp(a)-associated lipids was tested ex vivo in primary human monocytes by RNA sequencing, ELISA, Western blot, and transendothelial migratory assays. Using immunofluorescence staining and single-cell RNA sequencing, the phenotype of macrophages was investigated in human atherosclerotic lesions.

**RESULTS:**

Compared with healthy individuals with low/normal Lp(a) levels (median, 7 mg/dL [18 nmol/L]; n=13), individuals with elevated Lp(a) levels (median, 87 mg/dL [218 nmol/L]; n=12) demonstrated an increase in lipid species, particularly diacylglycerols (DGs) and lysophosphatidic acid (LPA). DG and the LPA precursor lysophosphatidylcholine were enriched in the Lp(a) fraction. Ex vivo stimulation with DG(40:6) demonstrated a significant upregulation in proinflammatory pathways related to leukocyte migration, chemotaxis, NF-κB (nuclear factor kappa B) signaling, and cytokine production. Functional assessment showed a dose-dependent increase in the secretion of IL (interleukin)-6, IL-8, and IL-1β after DG(40:6) and DG(38:4) stimulation, which was, in part, mediated via the NLRP3 (NOD [nucleotide-binding oligomerization domain]-like receptor family pyrin domain containing 3) inflammasome. Conversely, LPA-stimulated monocytes did not exhibit an inflammatory phenotype. Furthermore, activation of monocytes by DGs and LPA increased their transendothelial migratory capacity. Human atherosclerotic plaques from patients with high Lp(a) levels demonstrated colocalization of Lp(a) with M1 macrophages, and an enrichment of CD68^+^IL-18^+^TLR4^+^ (toll-like receptor) TREM2^+^ (triggering receptor expressed on myeloid cells) resident macrophages and CD68^+^CASP1^+^ (caspase) IL-1B^+^SELL^+^ (selectin L) inflammatory macrophages compared with patients with low Lp(a). Finally, potent Lp(a)-lowering treatment (pelacarsen) resulted in a reduction in specific circulating DG lipid subspecies in patients with cardiovascular disease with elevated Lp(a) levels (median, 82 mg/dL [205 nmol/L]).

**CONCLUSIONS:**

Lp(a)-associated DGs and LPA have a potential role in Lp(a)-induced monocyte inflammation by increasing cytokine secretion and monocyte transendothelial migration. This DG-induced inflammation is, in part, NLRP3 inflammasome dependent.

**Highlights**

- This study demonstrates that healthy individuals with elevated levels of lipoprotein(a) have a distinct lipidome characterized by elevated diacylglycerol (DG) and lysophosphatidic acid levels.
- DGs are enriched in the lipoprotein(a) fraction.
- DGs induce a proinflammatory gene signature in primary human monocytes.
- DGs are able to induce monocyte activation, as attested by the secretion of the proinflammatory cytokines IL (interleukin)-6, IL-8, and IL-1β and increased transendothelial migration capacity.
- The DG-induced increase in proinflammatory response in monocytes is partially NLRP3 (NOD [nucleotide-binding oligomerization domain]-like receptor family pyrin domain containing 3) inflammasome dependent.

Mendelian randomization and epidemiology studies have established lipoprotein(a) (Lp[a]) as an independent and likely causal risk factor for cardiovascular disease (CVD).^1–3^ Individuals with elevated Lp(a) levels are hallmarked by increased arterial wall inflammation (as assessed by FDG-PET/CT [fludeoxyglucose-18-positron emission tomography/computed tomography]), accompanied by enhanced accumulation of peripheral blood mononuclear cells in the arterial wall compared with individuals with normal Lp(a) levels (7 [2–28] mg/dL).^4^ Further investigation in the mechanisms underlying this increased arterial wall inflammation revealed that monocytes obtained from individuals with elevated Lp(a) levels exhibit an activated and inflammatory phenotype, as well as increased transendothelial migratory capacity.^4^ Furthermore, exposure of human arterial endothelial cells (HAECs) to Lp(a) at a concentration of 100 mg/dL resulted in endothelial cell (EC) inflammation and increased leukocyte extravasation through the upregulation of PFKFB3 (6-phosphofructo-2-kinase/fructose-2,6-biphosphatase 3)-mediated inducible glycolysis.^5^ The induction of these atherogenic pathways triggered in monocytes and ECs has mainly been attributed to the proinflammatory oxidized phospholipids (OxPLs) associated with the apo(a) moiety of Lp(a).^4,5^ Using recombinant apo(a) 17 kringle constructs with and without OxPL-binding sites (17 kringle ΔLBS [lysine binding site]) or murine monoclonal antibody E06 binding OxPL, it was substantiated that OxPLs are essential for the induction of TNFα (tumor necrosis factor alpha) and IL (interleukin)-6 secretion.^4^

The term OxPL encompasses a large number (tens to hundreds) of species (oxidation-specific epitopes) that can be generated by enzymatic and nonenzymatic oxidation of phosphocholine-based phospholipids.^6^ OxPLs are established as significant players in Lp(a)-induced atherosclerosis, as well as the initiation and progression of aortic valve stenosis.^7–9^ In aortic valve stenosis, the Lp(a)-induced pathogenic processes were additionally attributed to the high content of downstream products of unoxidized phosphatidylcholine and OxPL, namely lysophosphatidylcholine (LPC). LPC can be converted into lysophosphatidic acid (LPA) by ATX (autotaxin), indicating that the downstream products of OxPLs also exert adverse biological effects.^10,11^ Nevertheless, Lp(a) is a complex lipid-rich particle, where additional lipid species could also function as proatherogenic mediators. Lipids are crucial in cellular processes by serving as building blocks for cell membranes and providing energy.^12,13^ Moreover, lipids can function as secondary messengers, which are pivotal for mediating signal transduction. Hence, it is important to delve deeper into the identification of distinct Lp(a)-derived lipid species that are associated with CVD and elucidate their respective functions.

We conducted a lipidomics analysis on both complete plasma and purified lipoprotein fractions obtained from healthy individuals with either elevated or low/normal levels of Lp(a). Moreover, the atherogenicity of the lipid species enriched on Lp(a) was assessed by investigating the inflammatory response of monocytes and ECs, as well as the capacity of monocytes to migrate through the endothelium. We validated our findings in vivo by immunofluorescence staining and single-cell RNA sequencing (scRNA-seq) of atherosclerotic plaques from patients with either high or low Lp(a). Finally, we investigated whether specific and potent Lp(a) lowering by apo(a) antisense (pelacarsen) therapy can alter the plasma lipidome of patients with CVD with elevated Lp(a) levels.

## MATERIALS AND METHODS

### Study Population

Plasma samples were obtained from healthy individuals with normal Lp(a) (median, 7 mg/dL [17.5 nmol/L]; n=13), healthy individuals with elevated Lp(a) (median, 87 mg/dL [217.5 nmol/L]; n=12; 2017–2018), and the phase 2b pelacarsen study (URL: https://www.clinicaltrials.gov; unique identifier: NCT03070782; 2017–2018), where patients with CVD received pelacarsen treatment for 26 weeks. The exclusion and inclusion criteria and the study design have been described in detail previously.^14,15^ In brief, Lp(a) plasma levels of ≥50 mg/dL (125 nmol/L) were defined as elevated. In the healthy observational cohort, individuals with elevated Lp(a) (median, 87 mg/dL [217.5 nmol/L]; n=12) were matched for age, sex, and body mass index to individuals with normal Lp(a) (median, 7 mg/dL [17.5 nmol/L]; n=13).^14^ The samples of the phase 2b pelacarsen study were obtained during a site-specific substudy at Amsterdam UMC (University Medical Center), locatie Academic Medical Centre. Fourteen patients with established CVD and elevated Lp(a) on standard-of-care preventative therapy for CVD risk factors (excluding Lp(a)) were included and randomized at Amsterdam UMC.^14^ The major exclusion criteria were acute coronary syndrome, major cardiac surgery, stroke, or transient ischemic attack within 6 months before screening, as well as revascularization, major noncardiac surgery, or lipoprotein apheresis within 3 months before screening.^15^ The study protocol was approved by the Medical Ethical Committee of the Amsterdam Medical Center in Amsterdam, the Netherlands. All volunteers provided written informed consent before enrollment.

### Biochemical Measurements

Blood was collected while patients were in a fasting state. Plasma total cholesterol, HDL (high-density lipoprotein) cholesterol, triglycerides, and Lp(a) levels were analyzed using commercially available methods. LDL (low-density lipoprotein) cholesterol levels were calculated using the Friedewald equation.

In the pelacarsen study, lipid profiles were measured with commercially available kits at Medpace Reference Laboratories (Leuven, Belgium), and Lp(a) molar concentrations, representing apo(a) particle number, were measured by an isoform-independent assay (Northwest Lipid Metabolism and Diabetes Research Laboratories, University of Washington).

### Lipidomics Analysis

Lipidomics analysis of samples was performed at the Core Facility Metabolomics of Amsterdam UMC, locatie Academic Medical Centre, Amsterdam, the Netherlands. All lipidomics readouts have been made available in Data Sets S1 through S3. Lipidomics analysis was performed as described previously by Herzog et al^16^ using normal-phase high-performance liquid chromatography–mass spectrometry in both positive and negative ionization modes. In this study, the high-performance liquid chromatography system consisted of an Ultimate 3000 binary high-performance liquid chromatography pump, a vacuum degasser, a column temperature controller, and an auto sampler (Thermo Scientific, Waltham, MA). The normal-phase system consisted of a Luna 2×250-mm silica 100 Å column with a 5-µm particle diameter (Phenomenex), and the column temperature was maintained at 25 °C. Phospholipids were separated from interfering compounds by a linear gradient between solution B (chloroform/methanol, 97:3 v/v) and solution A (methanol/water, 85:15 v/v). Solution A contained 0.125 mL of formic acid and 0.25 mL of 25% (v/v) aqueous ammonia per liter of eluent; solution B contained 0.125 mL of formic acid/L. The gradient (0.3 mL/min) was as follows: 0 to 1 minute, 10%A; 1 to 4 minutes, 10%A to 20%A; 4 to 12 minutes, 20%A to 85%A; 12 to 12.1 minutes, 85%A to 100%A; 12.1 to 14.0 minutes, 100%A; 14 to 14.1 minutes, 100%A to 10%A; and 14.1 to 15 minutes, equilibration with 10%A. All gradient steps were linear, and total analysis time, including the equilibration, was 15 minutes. Subsequently, a Q Exactive Plus (Thermo Scientific) mass spectrometer was used in the negative and positive electrospray ionization modes. In both ionization modes, mass spectra of the lipid species were obtained by continuous scanning from m/z 150 to m/z 2000 with a resolution of 280 000. Nitrogen was used as the nebulizing gas. The spray voltage used was 2500 V (−) and 3500 V (+), and the capillary temperature was 256 °C. S-lens radio frequency level, 50; auxiliary gas, 10; auxiliary gas temperature, 300 °C; sheath gas, 50; and sweep cone gas, 2.

### RNA Sequencing and Analyses: Study Cohorts

RNA-sequencing (RNA-seq) data were used from the healthy observational cohort and the phase 2b pelacarsen study, which has been described previously.^14^ These RNA-seq data are available from the corresponding author upon reasonable request. In brief, CD14^+^ monocytes were isolated from whole blood and lysed in TriPure Isolation Reagent (Roche). RNA was isolated, and the quality was assessed using the Bioanalyzer software. Subsequently, RNA-seq libraries were constructed using the NEBNext Ultra Directional RNA Library Prep Kit for Illumina (New England Biolabs; E7420) according to the manufacturer’s instructions. Total RNA was purified using the rRNA depletion kit (New England Biolabs; E6310), followed by cDNA synthesis and amplification of the libraries by real-time polymerase chain reaction. Lastly, sequencing was performed on Illumina cBot and HiSeq 4000 with 151-cycle paired-end flow cell lanes. Obtained reads were processed through the Illumina data analysis pipeline RTA (v2.7.7) and Bcl2fastq (v2.20) and were aligned to the human genome (version hg38) with STAR (v2.5.2b; RNA-seq) or HISAT2 (v2.1.0). Then, reads were filtered on MAPQ (mapping quality) ≥30 and were counted in exons and aggregated per gene using the HOMER (v4.9.1) analyzeRepeats.pl script. Differential expression of genes was assessed with DESeq2 (v1.22.2) in an R (v3.5.3) environment. *P*-adjusted values <0.1 were considered statistically significant. The R scripts are available on Zenodo and can be accessed at doi.org/10.5281/zenodo.8435690.

### Lipoprotein Isolation

The Lp(a) and LDL fractions were isolated from healthy volunteers with elevated Lp(a) levels. Each subject provided written informed consent, and blood was collected in EDTA-containing vacutainer tubes. Plasma was obtained by centrifugation (3800 rpm, 4 °C, 10 minutes).

Plasma density was adjusted by adding potassium bromide (KBr), then 3.5 mL plasma was added to 12.5 mL ultra-clear soft tubes (6× tubes per ultracentrifuge; Beckman Coulter, Inc, CA). Lp(a) and LDL fractions were isolated by KBr density gradient ultracentrifugation as described previously.^4^ In short, a discontinuous gradient was formed by layering 2 mL of d=1.225 g/mL KBr on top of the plasma, followed by 4 mL of d=1.100 g/mL KBr and subsequently 3 mL of d=1.006 g/mL KBr. Samples were centrifuged for 19 hours at 10 °C at 29 000 rpm without brake in a SW 41 Ti rotor (Beckman Coulter, Inc). The Lp(a) and LDL fractions were separated, sliced, and dialyzed using PBS. When Lp(a) was isolated for in vitro use, samples were filter sterilized (pore size, 0.2 µm; Sartorius, Göttingen, Germany) and concentrated using the Amicon Centrifugal Filter Units feature (10 000 MWCO [molecular weight cut off]; Millipore) and used directly after isolation. Lp(a) and LDL fractions for lipidomics were concentrated using the Amicon Centrifugal Filter Units feature (10 000 MWCO; Millipore) and subsequently purified further using fast-performance liquid chromatography. Subsequently, Lp(a) and apoB concentrations were measured using commercially available immunoturbidimetric enzymatic assays (Lp(a) 21 FS, catalog number: 1 7139 99 10 921; Diasys, Holzheim, Germany) on a Selectra system (Sopachem, Ochten, the Netherlands).

### Fast-Performance Liquid Chromatography

The Lp(a) and LDL ultracentrifugation fractions were further purified by fast-performance liquid chromatography using an ÄKTAexplorer 10S system (GE Healthcare, Lifesciences Division, Diegem). All individual ultracentrifugation fractions were diluted 1:1 with Tris/NaCl (50/150 mmol/L), and a 500-μL sample/buffer mixture was loaded onto a Superose 6 Increase size exclusion chromatographic column (GE Healthcare). The flow rate was 0.5 mL/min for 60 minutes for each run, and the elution profiles were monitored at 2 wavelengths: 214 and 280 nm. Subsequently, 250-μL fractions were collected in 96-well plates for each of the ultracentrifugation lipoprotein samples. Finally, the fractions corresponding with the elution time of the lipoproteins of interest, as established previously,^17^ were pooled and used for lipidomics assessment.

### Commercial Lipids

Diacylglycerol (DG; DG[18:0-20:4]; 1-stearoyl-2-arachidonoyl-*sn*-glycerol; 800818C-10 mg; referred to as 38:4), DG(18:0-22:6; 1-stearoyl-2-docosahexaenoyl-*sn*-glycerol; 800819C-10 mg; referred to as 40:6), and LPA(18:0; 1-stearoyl-2-hydroxy-sn-glycero-3-phosphate [sodium salt]; 857128P-25 mg) were bought from Avanti Polar Lipids and dissolved in DMSO (dimethyl sulfoxide; Alabama).

### Ex Vivo Monocyte Stimulation

Human peripheral blood mononuclear cells were isolated from buffy coats (Sanquin, Amsterdam, the Netherlands) by Ficoll density gradient centrifugations (Axis-Shield) as described previously.^18^ In brief, after washing peripheral blood mononuclear cell fraction, CD14^+^ monocytes were isolated using human CD14 magnetic beads and MACS cell separation columns according to the manufacturer’s instructions (Miltenyi Biotec, Leiden, the Netherlands). All experiments were conducted in RPMI 1640 (Gibco) supplemented with GlutaMAX, penicillin, and streptomycin. To determine the proinflammatory effects of DGs and LPA on monocytes, CD14^+^ monocytes were stimulated with 1-, 2-, 5-, 10-, or 20-μM DG(40:6), DG(38:4), or LPA for 24 hours for cytokine measurements and 1 or 10 DG(40:6), DG(38:4), or LPA for 4 hours for RNA-seq.

To block Lp(a)-OxPL signaling, 300 μg/mL E06 (mIgG; OXI010101; Oxitope Pharma BV, Naarden, the Netherlands) was preincubated with 100 mg/dL Lp(a)^5^ for 2 hours before adding the complexes to the monocyte culture. When stated, monocytes were stimulated with 1-, 2-, 5-, 10-, or 20-μM DG(40:6), DG(38:4), or LPA for 24 hours and DMSO or 10-μM DG(40:6), DG(38:4), or LPA for 4 hours for transcriptome analysis. To inhibit the NLRP3 (NOD [nucleotide-binding oligomerization domain]-like receptor family pyrin domain containing 3) inflammasome, 1 µM CRID3 (cytokine release inhibitory drug 3; Sigma-Aldrich, St. Louis, MO) was used to pretreat monocytes 1 hour before stimulation with 100 mg/dL Lp(a).

### Cell Culture

Primary HAECs (Lonza, Baltimore, MD) were seeded on fibronectin (10 μg/mL; Sigma-Aldrich)-coated tissue culture–treated flasks in EGM-2 (endothelial cell growth medium-2) medium (CC-3162; Lonza). For all experiments, HAECs between passages 6 and 8 were used. To determine the proinflammatory effects of DGs and LPA on ECs, HAECs were stimulated with 1-, 2-, 5-, 10-, or 20-μM DG(40:6), DG(38:4), or LPA for 24 hours.

### Cytokine Measurements

Cytokine secretion was measured in the supernatant of human monocytes using commercially available ELISA kits for IL-6, IL-8, IL-1β, MCP-1 (monocyte chemoattractant protein-1), and Casp1 (caspase-1) according to the manufacturer’s instructions (Invitrogen, CA or R&D Systems, Minneapolis, MN). High-binding half-area 96-well plates were used for the IL-6, IL-8, IL-1β, and MCP-1 ELISAs. To measure NLRP3 secretion in the supernatant of human monocytes, the human NLRP3 ELISA kit was used according to the manufacturer’s instructions (Wuhan Fine Biotech Co; EH4202). In brief, samples and standards were added according to the optimal dilutions and bound to the capture antibody. Subsequently, the secondary antibody and streptavidin-HRP (horseradish peroxidase) were added. Tetramethylbenzidine substrate solution was used for detection, and after adding stop solution, absorbance was measured at 490 and 570 nm using a VersaMax plate reader.

### Transendothelial Migration Assay

To mimic an endothelial inflammatory response, a monolayer of HAECs was stimulated with 1 ng/mL TNFα for 16 to 20 hours. Monocytes were either unstimulated (medium), DMSO (solvent control), 1- or 10-μM DG(40:6), DG(38:4), or LPA for 18 hours. HAEC medium was changed to regular EGM-2 medium 2 hours before the addition of monocytes. Next, the stimulated monocytes (1×10^5^ per well) were added to the ECs in a 12-well plate and incubated for 30 minutes at 37 °C at 5% CO_2_. Subsequently, the cells were fixed with 3.7% formaldehyde (Sigma-Aldrich) and washed with PBS. Then, 2 to 3 images were captured per well in triplicate using a Zeiss Axiovert 200 inverted microscope (Planapochromat 10×/0.45 M27 Zeiss objective; Carl Zeiss, Inc, Jena, Germany). To assess the adhesion and migration capacity of monocytes, 4 different donors were used. Transmigrated monocytes were distinguished from adherent cells by their transitions from bright to dark morphology.

### Immunoblotting

Stimulated monocytes were lysed using the RIPA (radioimmunoprecipitation assay) buffer (Thermo Fisher Scientific, Waltham, MA) containing protease and phosphatase inhibitors (Roche Diagnostics GmbH, Mannheim, Germany). Then, proteins were separated on 4% to 12% polyacrylamide gels (Thermo Fisher Scientific) in MOPS (3-(N-morpholino)propanesulfonic acid) running buffer (Life Technologies, Carlsbad, CA) by SDS-PAGE and transferred to nitrocellulose membranes (Bio-Rad, Hercules, CA). The membranes were blocked using 5% BSA in TBS-T (1X Tris-Buffered Saline, 0.1% Tween 20) and then incubated with primary antibodies against Casp1 (Cell Signaling Technologies, Danvers, MA; D7F10; 3866) and β-actin (GeneTex, Irvine, CA; GT5512). Subsequently, the membranes were incubated with secondary antibodies HRP-conjugated swine anti-rabbit (Dako, Santa Clara, CA; P0399) or rabbit anti-mouse immunoglobulins (Dako; P0260). Protein bands were visualized using SuperSignal West Pico PLUS Chemiluminescent Substrate or SuperSignal West Femto Maximum Sensitivity Substrate (Thermo Fisher Scientific) and imaged with ChemiDoc MP Imaging System (Bio-Rad).

### RNA-seq and Analyses: Ex Vivo Monocyte Experiment

This RNA-seq from the ex vivo monocyte experiments data has been made publicly available at the NCBI (National Center for Biotechnology Information) Gene Expression Omnibus and is accessible through the GEO Series accession number GSE249911. All differentially expressed genes from the ex vivo monocyte experiments are available in Tables S1 through S4. Human monocytes were lysed using TriPure Isolation Reagent (Roche, Basel, Switzerland) and stored at −80 °C before RNA isolation. RNA was isolated according to the manufacturer’s instructions, and the quality was determined using Lab-on-a-Chip RNA 6000 Nano on Agilent Bioanalyzer (Agilent Technologies, Santa Clara, CA). Library construction, quality control, and sequencing were performed at Novogene. In brief, mRNA was purified from total RNA using Poly T Oligo Attached Magnetic Beads. After fragmentation, the first-strand cDNA was synthesized using random hexamer primers, followed by the second-strand cDNA synthesis using either dUTP (2´-deoxyuridine, 5´-triphosphat) for directional library or dTTP for nondirectional library.^19^ The library was checked with Qubit and real-time polymerase chain reaction for quantification and bioanalyzer for size distribution detection. Quantified libraries were pooled and sequenced on a HiSeq instrument (Illumina) with 150-bp paired-end reads and 10 G data per sample. Obtained reads were processed through in-house Perl scripts at Novogene and were aligned to the human genome using Hisat2 (v2.0.5). Then, reads were counted with featureCounts (v1.5.0-p3).^20^ Figure S1 displays the principal component analysis plots. Differential expression of genes was assessed with DESeq2, R package (1.40.2). The resulting *P* values were adjusted using the Benjamini-Hochberg approach for controlling the false discovery rate. Pathway and gene ontology analyses were performed using clusterProfiler (4.8.2) and visualized with pathview (1.40.0). The targeted heatmap was visualized using pheatmap (1.0.12).

### Immunofluorescence Staining of Human Carotid Endarterectomy Plaques

The Athero-Express Biobank (UMC Utrecht, the Netherlands) is an ongoing prospective biobank including patients undergoing carotid and femoral endarterectomy. The design and sample processing have been reported previously.^16^ The plaque segments from patients with known Lp(a) levels have been included to perform immunofluorescence staining. First, plaque sections were dewaxed using xylene (3× 100%; 10 minutes) and dehydrated in decreasing concentrations of ethanol (2× 100%, 96%, and 70%; 1 minute). Then, heat-induced epitope retrieval was performed (121 °C, EDTA pH 8.0 or citrate pH 6.0 where appropriate) for 10 minutes followed by blocking with Ultravision Protein Block (Thermo Fisher Scientific) for 10 minutes at room temperature. The plaque sections were subsequently incubated with the primary antibody: MARCO (macrophage receptor with collagenous structure) rabbit anti-human (Abcam, Cambridge, United Kingdom; ab231046), CD68 mouse anti-human; IgG3 (Agilent Technologies; M0876), or LPA4 mouse anti-human; IgG1 (kind gift from Prof Tsimikas; EMD Millipore; MABS1284). Then, sections were washed and incubated for 30 minutes in the dark at room temperature with the secondary antibodies: GαRb (goat anti-rabbit) IgG Alexa 488 (Invitrogen, Waltham, MA; A11304), GαMs (goat anti-mouse) IgG1 Alexa 568 (Invitrogen, Waltham, MA; A21124), or GαMs IgG3 Alexa 647 (Jackson ImmunoResearch, PA; 115-605-209). Sections were washed and stained with Hoechst (Thermo Fisher Scientific; 62249) for 5 minutes at room temperature. The specificity of the primary antibodies was tested by using their isotype controls as a negative control. The isotype controls matched the host species and isotype class of the primary antibody and were used in the same concentration. The following isotype controls were used: Mouse IgG1, kappa Isotype Control (BD Biosciences; 349043) for LPA4, BV421 Mouse IgG3, kappa Isotype Control (BD Biosciences; 563314) for CD68, and Rabbit IgG control polyclonal antibody (Proteintech; 30000-0-ap) for MARCO. Sections were imaged using a Leica TCS SP8 confocal laser scanning microscope.

### Histological Staining

Frozen healthy aorta sections were cut at 7 µm and stained with hematoxylin (Mayer, Clin-Tech Limited, Guildford, United Kingdom) and eosin (in house). For the visualization of Lp(a), tissues were fixed with acetone (Sigma-Aldrich) for 10 minutes, followed by 10 minutes of blocking with Ultravision Protein Block (Thermo Fisher Scientific). The tissue was stained with apo(a) (LPA4, 1:25) for 1 hour, washed, and incubated with the secondary antibody (poly-HRP-anti-mouse IgG, undiluted; ImmunoLogic, Arnhem, NL) for 30 minutes. Apo(a) was visualized with DAB (3,3’diaminobenzidine; ImmunoLogic) and counterstained with hematoxylin. Sections were imaged on a Leica DM6B light microscope.

### scRNA-seq and Data Processing

The transcriptome of macrophages in human atherosclerotic lesions was investigated in plaques from patients with elevated Lp(a) (≥75 nmol/L, ≥30 mg/dL; n=10) and low Lp(a) (≤5.7 nmol/L, ≤2.28 mg/dL; n=10). These data are available from the corresponding author upon reasonable request. Processing of human plaques, single-cell library preparation and sequencing, and data analyses were performed identical to the study by Depuydt et al.^21^ Briefly, human carotid plaques were collected during carotid endarterectomy, cut, digested, and washed. Viable cells were FACS (fluorescence-activated cell sorting) sorted into 384-well plates and lysed. Then, cDNA was constructed using the SORT-seq protocol, and libraries were generated using the CEL-Seq2 protocol. Illumina libraries were generated by introducing a unique molecular identifier, barcode, and adaptor sequence. Libraries were sequenced paired end at 75-bp read length on Illumina NextSeq 500. Data were analyzed in an R 4.2.1 environment using Seurat 4.1.1.^22^ In brief, mitochondrial and ribosomal genes were excluded. In addition, doublets and low-quality cells were filtered out by including cells expressing 500 to 10 000 genes and genes expressed in a minimum of 3 cells. Using Seurat 4.1.1, the data were log normalized and scaled. Then, cell types were assigned to clusters by evaluating gene expression. Further subclustering was performed using canonical correlation analysis. False discovery rate was applied to correct for multiple testing. R scripts are available on GitHub and can be accessed at https://github.com/AtheroExpress/MicroanatomyHumanPlaque_scRNAseq. Data are visualized in tSNE (t-distributed stochastic neighbor embedding) plots and violin plots using the R script in the Supplemental Material (code: scRNAseq plots).

### Statistical Analysis

Lipidomics statistical analysis was conducted as described previously by Herzog et al.^16^ The raw high-performance liquid chromatography–mass spectrometry data were converted to mzXML format using MSConvert.^23^ Subsequently, the data set was processed using an in-house–developed metabolomics pipeline written in the R programming language (http://www.r-project.org). In brief, it consisted of the following steps: (1) preprocessing using the R package XCMS^24^ with minor changes to some functions to better suit the Q Exactive data; notably, the definition of noise level in centWave was adjusted and the step size in fillPeaks. The R code is available in the Supplemental Material (Pipeline Lipidomics Analysis); (2) identification of metabolites using an in-house database of phospholipids, with known internal standards indicating the position of most of the lipid clusters, matching m/z values within 3-ppm deviation; (3) isotope correction to obtain deconvoluted intensities for overlapping peak groups; (4) normalization on the intensity of the internal standard for lipid classes for which an internal standard was available and scaling on measured protein content per sample; (5) total value is the sum of the relative abundance of all identified lipid species; (6) a Student *t* test with post hoc Bonferroni correction was used for statistical comparison between the 2 groups.

For the ex vivo monocyte stimulation, data were analyzed using Graphpad Prism 8 (La Jolla, CA). Grubbs outlier tests were performed to identify outliers. This was followed by a Shapiro-Wilk test to assess for normality. When data followed a normal distribution, an ordinary 1-way ANOVA was performed followed by the Dunnett multiple comparisons test (compare the mean of each column with the mean of one column) or the Tukey multiple comparisons test (compare the mean of each column with the mean of every other column). When the data were not normally distributed, a Kruskal-Wallis test was performed followed by a Dunn multiple comparisons test. For the phase 2b pelacarsen study, cytokine secretion data were analyzed using a 2-tailed paired *t* test.

## RESULTS

### Blocking OxPL-Lp(a) Results in Residual IL-8 and IL-1β Secretion by Human Monocytes

**Figure 1.**
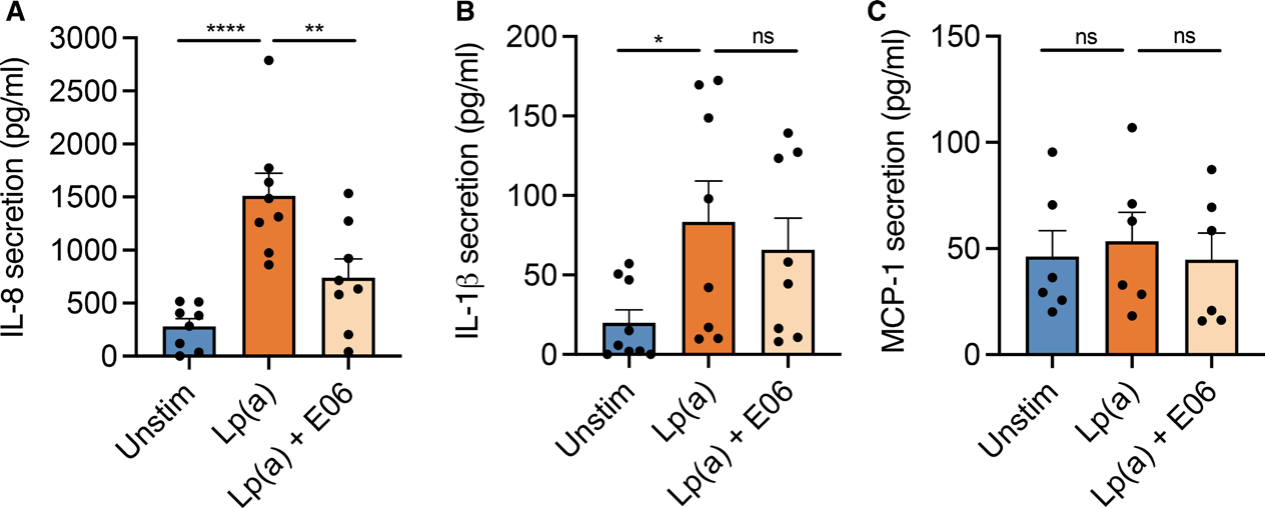
**Blocking oxidized phospholipid (OxPL)–lipoprotein(a) (Lp[a]) results in residual IL (interleukin)-8 secretion in monocytes.** CD14^+^ monocytes were stimulated 100 mg/dL Lp(a) in the presence or absence of E06 and Lp(a) for 24 hours. **A**, Secretion of IL-8 in the cell medium (n=8). *P*<0.0001 for unstimulated (Unstim) vs Lp(a) and *P*=0.0063 for Lp(a) vs Lp(a)+E06. **B**, IL-1β secretion in the cell medium (n=9). *P*=0.0416 for Unstim vs Lp(a) and *P*>0.9999 for Lp(a) vs Lp(a)+E06. **C**, Secretion of MCP-1 (monocyte chemoattractant protein-1) in the cell medium (n=6). No statistically significant difference between the conditions. Grubbs outlier tests were performed to identify outliers. This was followed by a Shapiro-Wilk test to test for normality. When data followed a normal distribution, an ordinary 1-way ANOVA was performed followed by Dunnett multiple comparisons test. When the data were not normally distributed, a Kruskal-Wallis test was performed followed by a Dunn multiple comparisons test (IL-1β secretion). *P<0.05, **P<0.01, ****P<0.0001.

To evaluate the inflammatory potential of Lp(a)-derived proinflammatory mediators, besides its known OxPLs, we explored to what extent blocking OxPLs could decrease Lp(a)-induced inflammation in monocytes. Monocytes derived from healthy volunteers were exposed to a physiologically relevant concentration of 100 mg/dL Lp(a)^5^ in the presence or absence of the OxPL-blocking antibody E06 (300 µg/mL). Lp(a) stimulation resulted in increased secretion of IL-8 (*P*<0.0001) and IL-1β (*P*=0.0416; Figure 1A and 1B). Although E06 partially blocked Lp(a)-induced IL-8 secretion (*P*=0.0063), there was still residual inflammation as shown by substantial IL-8 secretion (Δcytokine secretion, 456 pg/mL; 37%; Figure 1A). However, blocking OxPLs only minimally impacted Lp(a)-induced IL-1β secretion (*P*>0.9999; Figure 1B). Lp(a) stimulation of monocytes did not affect MCP-1 secretion (Figure 1C). While blocking OxPLs has a profound effect on reducing Lp(a)-induced monocyte IL-8 secretion, the remaining IL-1β secretion strongly suggests that additional Lp(a)-derived proinflammatory mediators contribute to Lp(a)-induced inflammation.

### Subjects With Elevated Lp(a) Exhibit a Distinct Lipidome Characterized by Increased Levels of DGs and LPA

**Figure 2.**
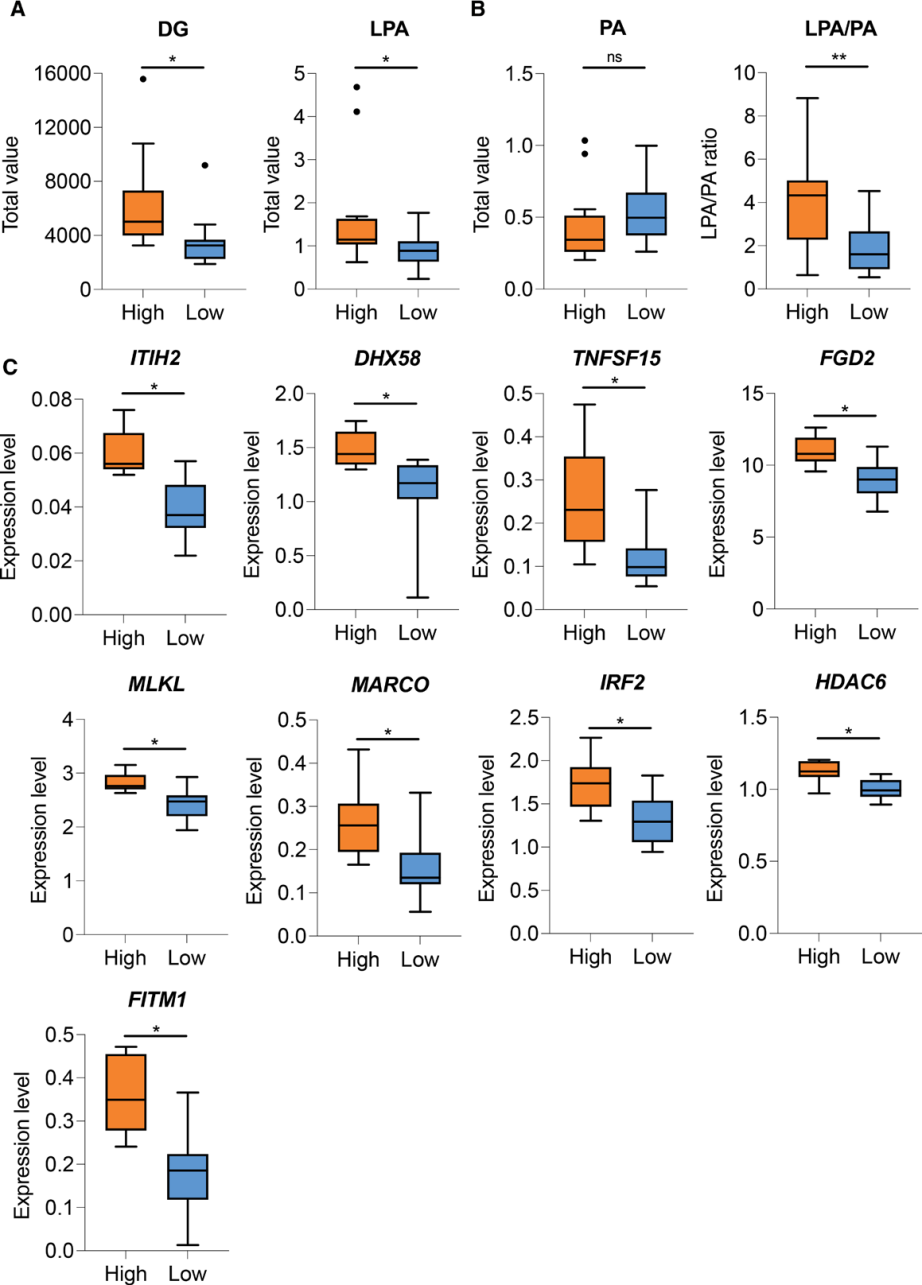
**Subjects with elevated lipoprotein(a) (Lp[a]) exhibit a distinct lipidome characterized by increased levels of diacylglycerols (DGs) and lysophosphatidic acid (LPA).** Boxplots demonstrating the total value of DG, LPA (**A**), phosphatidic acid (PA), and lysoPA/PA (**B**) ratio in healthy individuals with high levels of Lp(a) (orange, n=12) and low levels of Lp(a) (blue, n=13). Total value is the sum of the relative abundance of all identified lipid species. A Student *t* test with post hoc Bonferroni correction was used for statistical comparison between the 2 groups. Whiskers: Tukey (**C**) box plots of differentially expressed genes involved in leukocyte migration, T-cell activation, inflammation, and lipid droplet formation in monocytes from healthy individuals with high levels of Lp(a) (orange, n=10) and low levels of Lp(a) (blue, n=13). *P*-adjusted values <0.1 were considered statistically significant. *P<0.05, **P<0.01.

To identify potential additional players involved in this observed Lp(a)-induced residual inflammatory response, we conducted plasma lipidomics analysis on our cohort recently described by Stiekema et al.^14^ In this cohort, none of the individuals had a history of CVD and none of the subjects used medication. Twelve healthy individuals with high Lp(a) levels (median, 87 mg/dL [217.5 nmol/L]) were age, sex, and body mass index matched to 13 healthy individuals with low/normal Lp(a) levels (median, 7 mg/dL [17.5 nmol/L]; Table). Lipidomics analysis was performed for 25 major lipid classes (Figure S2), including cholesterol esters, phosphatidylcholines, and DGs. This analysis demonstrated that individuals with high Lp(a) levels have a higher plasma abundance of DG and LPA compared with individuals with low Lp(a) levels (Figure 2A). As LPA can be converted into phosphatidic acid (PA) and eventually DG, we also investigated the relative abundance of PA and the LPA/PA ratio (Figure 2B).^25,26^ Where the relative abundance of PA did not differ between individuals with low and high levels of Lp(a), the LPA/PA ratio was significantly increased in individuals with high Lp(a) levels (*P*=0.0012), demonstrating an altered plasma lipidome in individuals with elevated levels of Lp(a). This altered plasma lipidome was accompanied by a proinflammatory monocyte phenotype marked by the upregulation of genes involved in immune cell activation, *DHX58*(*P*=0.022), leukocyte migration, *ITIH2* (*P*=0.015), immune cell regulation via cytoskeleton-dependent membrane rearrangements, *FGD2* (*P*=0.038), and inflammation, including *TNFSF15* (*P*=0.036), *MLKL* (*P*=0.048), *MARCO* (*P*=0.050), *IRF2* (*P*=0.050), *HDAC6* (*P*=0.050), as well as *FITM1* (*P*=0.026), which is involved in lipid droplet formation (Figure 2C).

**Table.**
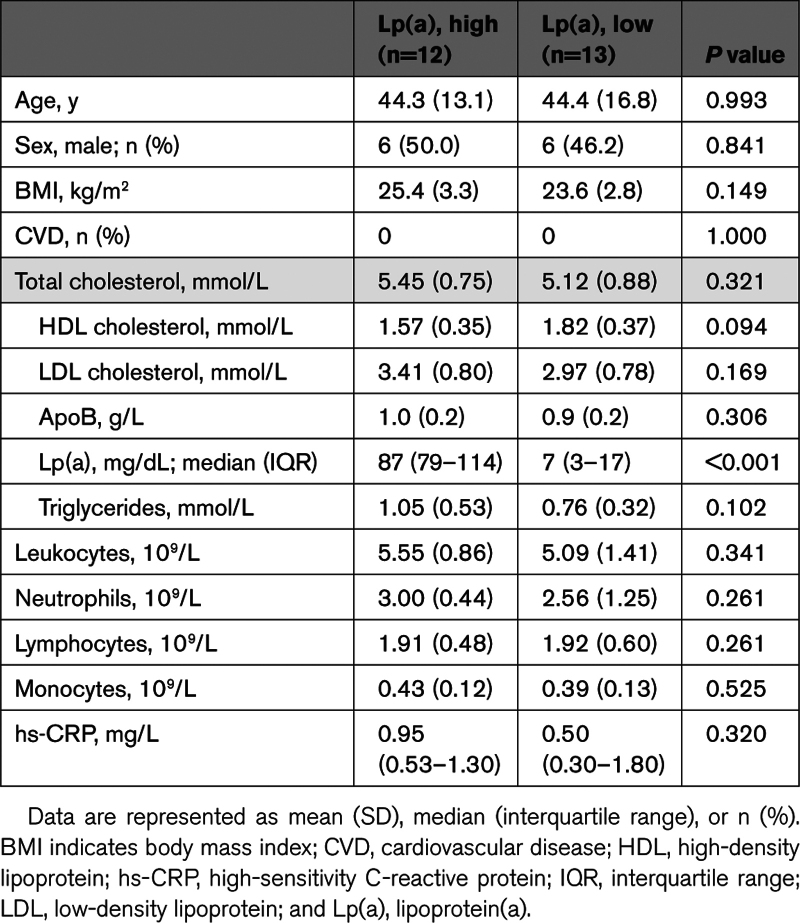
Baseline Characteristics in Healthy Individuals With Low and High Levels of Lp(a)

### Lp(a) Fractions Are Enriched With DGs

**Figure 3.**
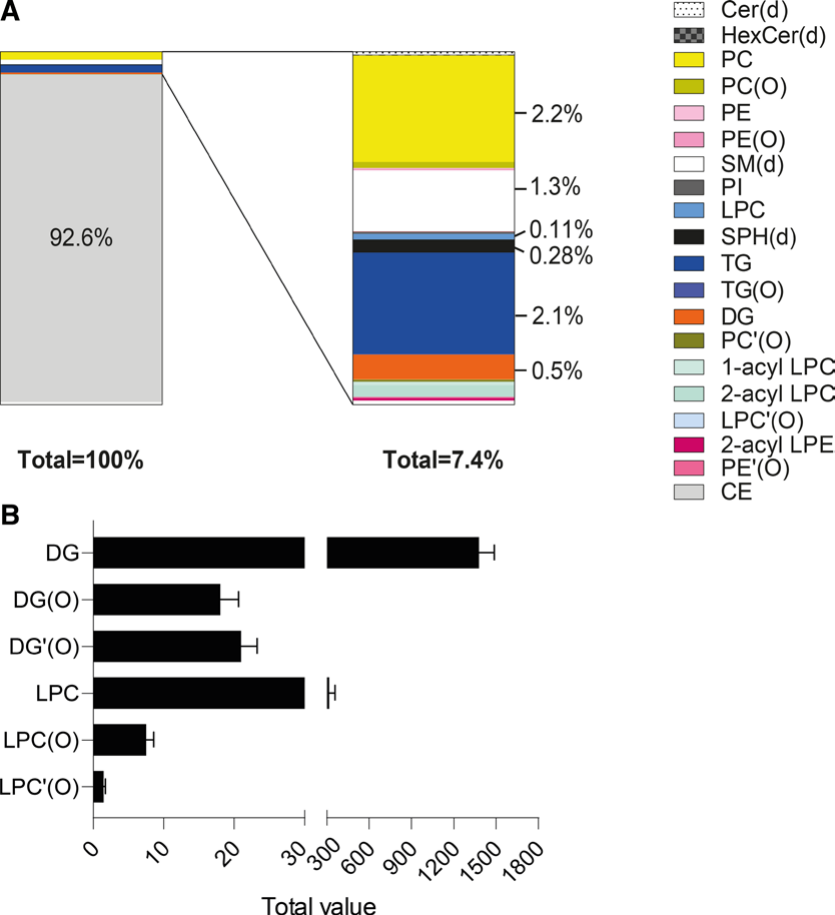
**Lipoprotein(a) (Lp[a]) fractions are enriched with diacylglycerols (DGs). A**, Per major lipid class, the mean percentage in the total lipidome of the Lp(a) fraction. **B**, The presence of DGs and the lysophosphatic acid precursor lysophosphatidylcholine (LPC) on Lp(a). Total value is the sum of the relative abundance of all lipid species of that lipid class. 1/2-acyl LPC indicates 1/2-lysophosphatidylcholine; 2-acyl LPE, 2-lysophosphatidylethanolamine; CE, cholesterol ester; Cer(d), ceramide; DG(O)/DG’(O), alkyl/alkenyl-alcyl glycerol; HexCer(d), hexosylceramide; LPC(O)/LPC’(O), alkyl/alkenyllysophosphatidylcholine; PC, phosphatidylcholine; PC(O)/PC’(O), alkyl/alkenylphosphatidylcholine; PE, phosphatidylethanolamine; PE(O)/PE’(O), alkyl/alkenylphosphatidylethanolamine; PI, phosphatidylinositol; SM(d), sphingomyelin/ceramide phosphocholine; SPH(d), sphinganine; TG, triacylglycerol; and TG(O), alkyl diacylglycerol.

Lipoproteins have been shown to have a distinct lipid profile, thereby altering not only the composition but also the function of the lipoprotein particle.^27–30^ Therefore, we investigated whether the observed increase in plasma DGs and LPA levels was related to an enrichment of DGs and LPA in the Lp(a) fraction. To study this, Lp(a) fractions were first isolated from healthy volunteers with elevated Lp(a) levels (>50 mg/dL) and subsequently purified by fast-performance liquid chromatography. Lipidomics analysis identified the presence of several major lipid classes in the Lp(a) fraction (Figure 3A; Figure S3C), with cholesterol ester being the predominant lipid class (mean, 92.6%; Figure 3A). Following cholesterol ester, the 5 main lipid classes in the Lp(a) fractions were phosphatidylcholine (mean, 2.2%), triacylglycerol (mean, 2.1%), ceramide phosphocholines (mean, 1.3%), DG (mean, 0.5%), and sphingomyelin (mean, 0.28%). The proportion of DGs (mean, 0.5%) within the Lp(a) fraction surpassed that observed in the LDL fraction (mean, 0.14%; Figure S4), implying that Lp(a) potentially serves as the preferential carrier of DGs compared with LDL. Besides Lp(a)-associated DGs, ether lipid analogues alkyl-acyl glycerol and alkenyl-acyl glycerol were also present in the Lp(a) fraction, albeit to a lesser extent (Figure 3B). In addition, the relative abundance of DGs enriched in Lp(a) fractions does differ per DG lipid subspecies, meaning that DG(P:Q) could have a higher relative abundance than DG(X:Y) in the Lp(a) fraction (Figure S3A). Interestingly, LPA was not observed in the purified Lp(a) fractions. However, LPC—the precursor of LPA—was observed in the Lp(a) fractions (mean, 0.11%; Figure 3A and 3B), a finding that is in line with previous studies, demonstrating that LPC is present in Lp(a).^11,31^ Similar to DGs, the relative abundance differed per LPC lipid species (Figure S3B).

### DGs Induce Monocyte Inflammation via NOD-Like Receptor, NF-κB, Toll-Like, and TNF Signaling

**Figure 4.**
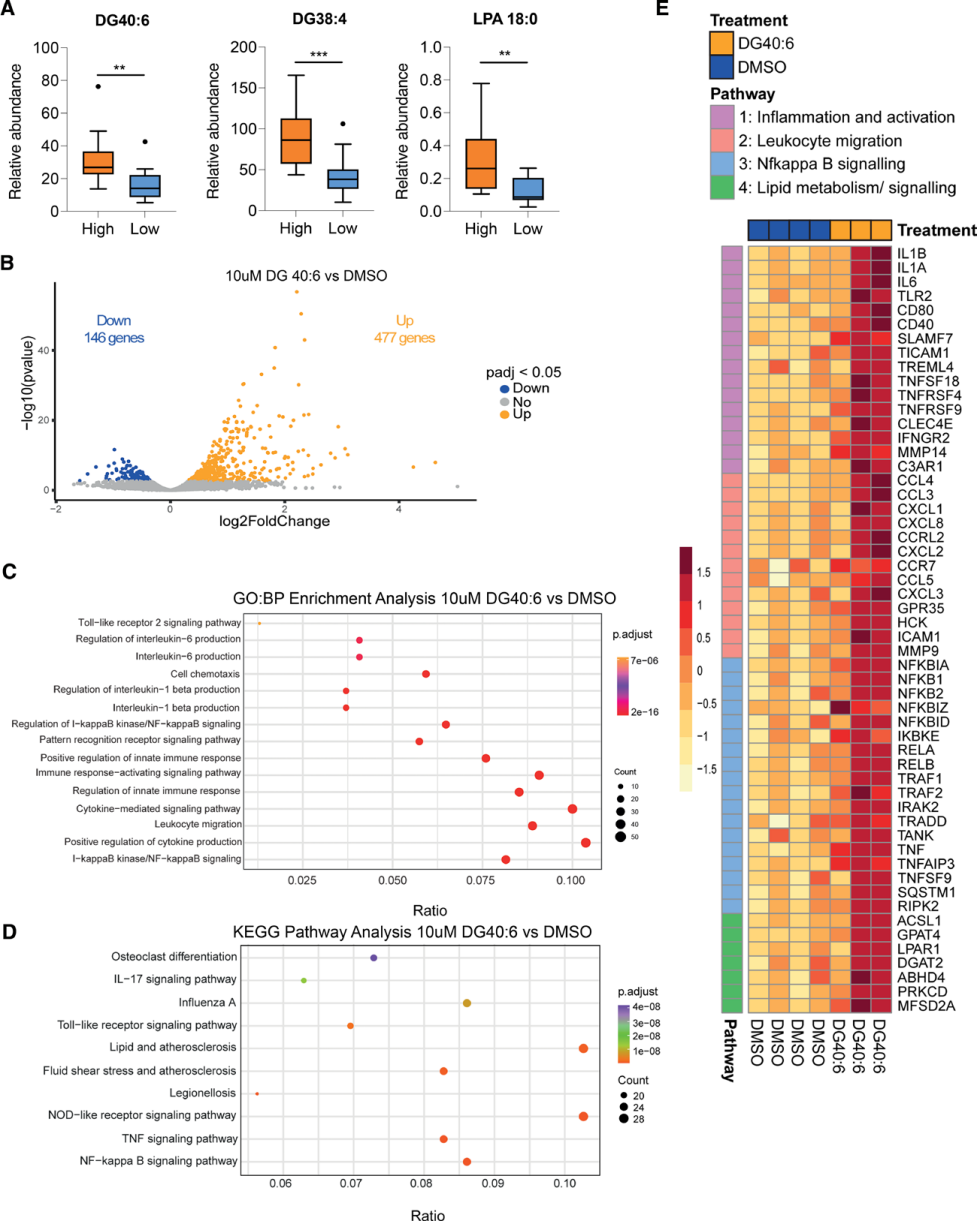
**Diacylglycerols (DGs) induce a proinflammatory phenotype in monocytes. A**, Boxplots demonstrating the relative abundance of DG(40:6), DG(38:4), and lysophosphatidic acid (LPA) in the plasma of healthy individuals with elevated lipoprotein(a) (Lp[a]) levels. A Student *t* test with post hoc Bonferroni correction was used for statistical comparison between the 2 groups. **B**, Volcano plot showing significantly differentially expressed genes in DG(40:6)-stimulated human primary monocytes adjusted for multiple testing using the Benjamini and Hochberg approach. Gene ontology (**C**) and KEGG (Kyoto Encyclopedia of Genes and Genomes) pathway analysis (**D**). **E**, Targeted heatmap displaying significantly upregulated genes. BP indicates biological process; DMSO, dimethyl sulfoxide; GO, gene ontology; IL, interleukin; NF, nuclear factor; NOD, nucleotide-binding oligomerization domain; and TNF, tumor necrosis factor. **P<0.01, ***P<0.001.

To investigate the potential impact of DG and LPA plasma levels on promoting an increased proinflammatory monocyte phenotype in individuals with elevated Lp(a) levels, we conducted a functional proof-of-concept study using commercially available DGs and LPA. The lipid species DG(38:4; 18:0-20:4; 1-stearoyl-2-arachidonoyl-*sn*-glycerol), DG(40:6; 18:0-22:6; 1-stearoyl-2-docosahexaenoyl-*sn*-glycerol), and LPA(18:0) were selected, as these lipid species are significantly increased in individuals with high levels of Lp(a) (*P*=0.0007, *P*=0.0067, and *P*=0.0044, respectively) and were commercially available (Figure 4A; Figure S5). Transcriptomic analysis was performed on DG- and LPA-stimulated primary human monocytes. Exposure to DG(40:6) resulted in 623 significantly differentially expressed genes in human monocytes, of which 477 genes were upregulated and 146 genes were downregulated (Figure 4B). Gene ontology analysis showed a significant upregulation in the proinflammatory pathway related to chemotaxis, leukocyte migration, NF-κB (nuclear factor kappa B) signaling, as well as cytokine production and regulation (Figure 4C; Table S1). In addition, the KEGG (Kyoto Encyclopedia of Genes and Genomes) pathway analysis demonstrated an enrichment in genes involved in NOD-like receptor, NF-κB, toll-like receptor, and TNF signaling (Figure 4D; Table S2). Lastly, Figure 4E displays a targeted heatmap of significantly upregulated genes. After adjusting for multiple testing, *AC233968.1* was significantly (*P*=0.0012) upregulated after DG(38:4) stimulation and *RPL7AP6* (*P*=4.20×10^−6^) after LPA stimulation (Tables S3 and S4).

### DGs Induce a Dose-Dependent Proinflammatory Response in Monocytes

**Figure 5.**
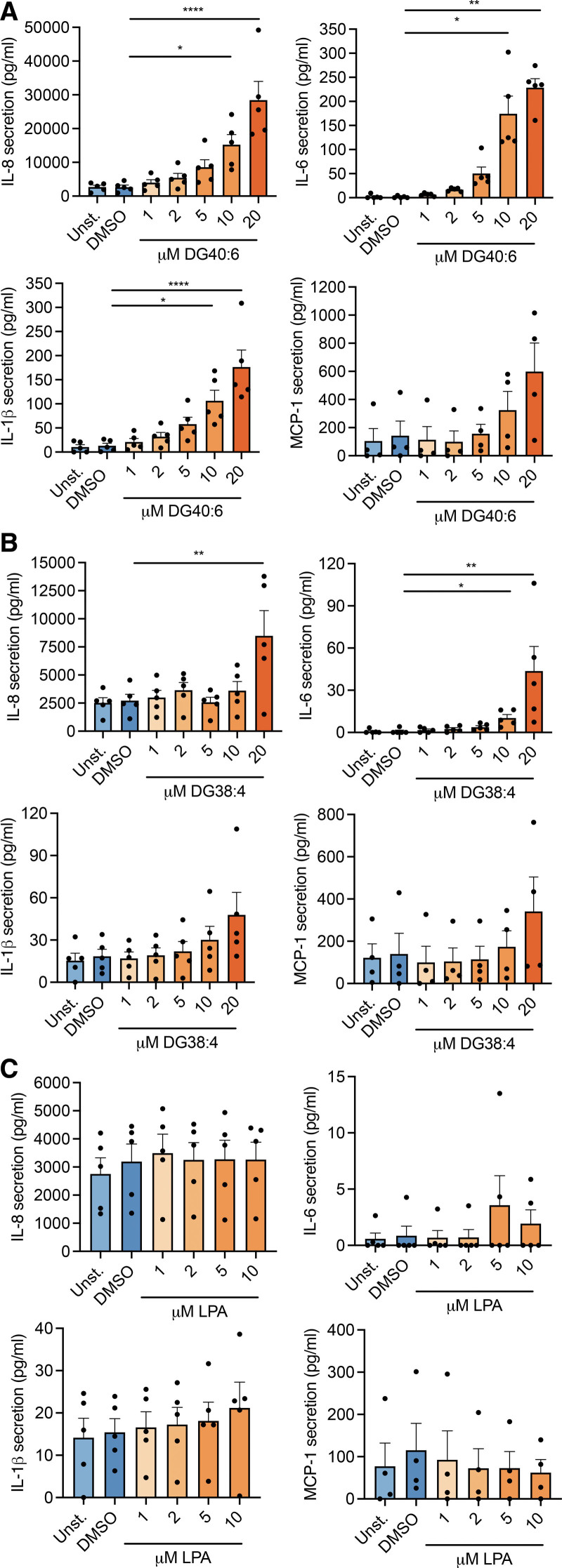
**Diacylglycerols (DGs) induce a dose-dependent proinflammatory response in monocytes. A**, IL (interleukin)-6, IL-8, IL-1β, and MCP-1 (monocyte chemoattractant protein-1) cytokine secretion in cell medium of monocytes stimulated with increasing concentration of DG(40:6; 24-h stimulation; n=5). **B**, IL-6, IL-8, IL-1β, and MCP-1 cytokine secretion in cell medium of monocytes stimulated with increasing concentration of DG(38:4; 24-h stimulation; n=5). **C**, IL-6, IL-8, IL-1β, and MCP-1 cytokine secretion in cell medium of monocytes stimulated with increasing concentration of lysophosphatidic acid (24-h stimulation; n=5). All data are mean±SEM. Grubbs outlier tests were performed to identify outliers. This was followed by a Shapiro-Wilk test to test for normality. When data followed a normal distribution, an ordinary 1-way ANOVA was performed followed by Tukey multiple comparisons test. When the data were not normally distributed, a Kruskal-Wallis test was performed followed by a Dunn multiple comparisons test. DMSO indicates dimethyl sulfoxide; and Unst, unstimulated. *P<0.05, **P<0.01, ****P<0.0001.

To investigate whether this proinflammatory gene signature translates into functional consequences, primary human monocytes derived from healthy individuals were stimulated with increasing concentrations of DG(38:4), DG(40:6), or LPA(18:0). Monocytes stimulated with DG(40:6) exhibited a dose-dependent increase in IL-8 (DMSO versus 10 μM, *P*=0.0306; DMSO versus 20 μM, *P*<0.0001), IL-6 (DMSO versus 10 μM, *P*=0.0100; DMSO versus 20 μM, *P*=0.0019), and IL-1β cytokine secretion (DMSO versus 10 μM, *P*=0.0112; DMSO versus 20 μM, *P*<0.0001; Figure 5A). MCP-1 secretion was not affected by DG(40:6) stimulation (Figure 5A), a finding that is consistent with our earlier finding, where we did not observe an increase in monocyte-derived MCP-1 secretion upon Lp(a) stimulation (Figure 1C). In line with DG(40:6), DG(38:4) stimulation resulted in a similar, albeit less potent dose-dependent secretion of IL-8 (DMSO versus 20 μM, *P*=0.0071) and IL-6 (DMSO versus 10 μM, *P*=0.0352; DMSO versus 20 μM, *P*=0.0021; Figure 5B). While IL-1β secretion was increased at the higher DG(38:4) concentrations, this increase was not statistically significant (unstimulated versus 20 μM, *P*=0.1295; DMSO versus 20 μM, *P*=0.2115). Similar to DG(40:6), MCP-1 secretion was not altered after exposure to DG(38:4). While LPA was enriched in the Lp(a) fraction, it was unable to elicit an inflammatory response in monocytes (Figure 5C). As both monocytes and ECs are important players in driving atherosclerosis progression, HAECs were stimulated with increasing concentrations of DG(38:4), DG(40:6), or LPA(18:0). Although DGs lead to monocyte activation, DG(40:6; Figure S6A), DG(38:4; Figure S6B) and LPA (Figure S6C) were unable to activate HAECs, as attested by the lack of changes in IL-6 and IL-8 at both the transcriptional and protein levels.

### DGs Enriched in the Lp(a) Fraction Activate the Inflammasome

**Figure 6.**
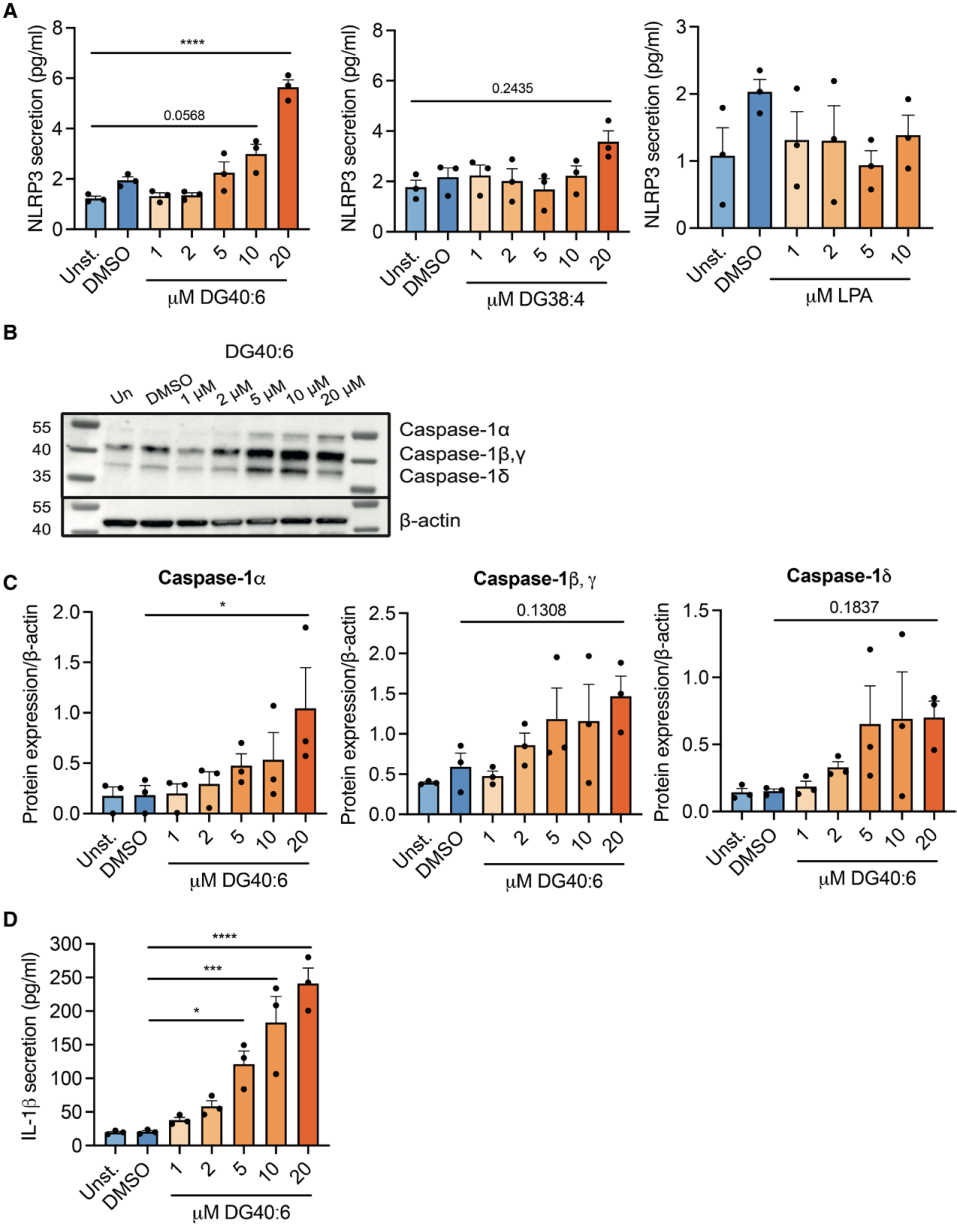
**Diacylglycerols (DGs) enriched in the lipoprotein(a) (Lp[a]) fraction activate the inflammasome. A**, NLRP3 (NOD [nucleotide-binding oligomerization domain]-like receptor family pyrin domain containing 3) secretion in cell medium of monocytes stimulated with increasing concentration of DG(40:6), DG(38:4), or lysophosphatidic acid (LPA; 24-h stimulation; n=3). **B**, Representative immunoblot demonstrating a dose-dependent increase in Casp1 (caspase-1) expression in monocytes after 24-hour DG(40:6) stimulation. **C**, Quantification of the immunoblot (n=3). **D**, IL-1β secretion in cell medium (n=3). All data are mean±SEM. Grubbs outlier tests were performed to identify outliers. This was followed by a Shapiro-Wilk test to test for normality. Then, the data were analyzed using an ordinary 1-way ANOVA followed by Dunnett multiple comparisons test or Tukey multiple comparisons test. DMSO indicates dimethyl sulfoxide; and Unst, unstimulated. *P<0.05, ***P<0.001, ****P<0.0001.

**Figure 7.**
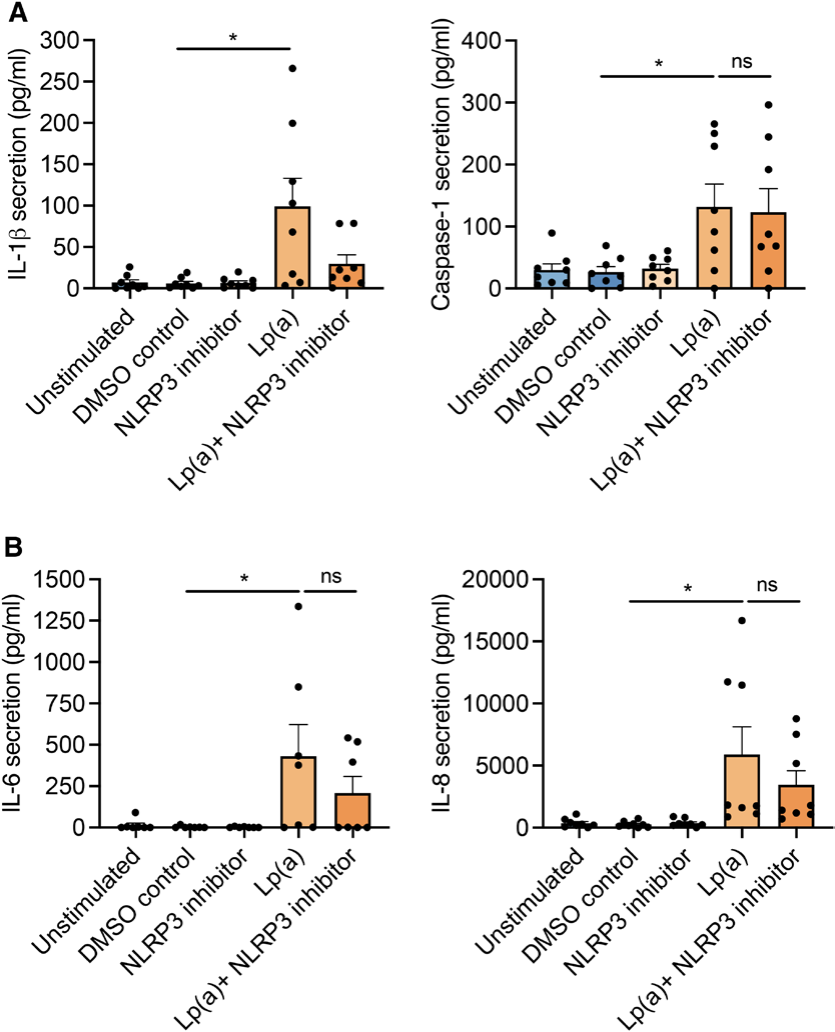
**NLRP3 (NOD [nucleotide-binding oligomerization domain]-like receptor family pyrin domain containing 3) inhibition can partly diminish lipoprotein(a) (Lp[a])-induced inflammation.** Monocytes were pretreated with 1 µM CRID3 (cytokine release inhibitory drug 3) or DMSO (dimethyl sulfoxide) control for 1 hour before stimulation with 1 mg/mL Lp(a) (n=7–8). IL (interleukin)-1β, Casp1 (caspase-1; **A**), IL-6, and IL-8 (**B**) secretion were measured in the cell medium. All data are mean±SEM. Grubbs outlier tests were performed to identify outliers. This was followed by a Shapiro-Wilk test to test for normality. When data followed a normal distribution, an ordinary 1-way ANOVA was performed followed by Dunnett multiple comparisons test. When the data were not normally distributed, a Kruskal-Wallis test was performed followed by a Dunn multiple comparisons test. *P<0.05.

Since DGs upregulated the NOD-like receptor signaling pathway and induced IL-1β secretion in monocytes, it was investigated whether the NLRP3 inflammasome pathway was underlying DG-induced inflammation in healthy monocytes. Indeed, DG(40:6) dose dependently increased NLRP3 secretion (DMSO versus 10 μM, *P*=0.0568; DMSO versus 20 μM, *P*<0.0001; Figure 6A). While NLRP3 secretion was increased at the higher DG(38:4) concentration, this increase was not statistically significant (DMSO versus 20 μM, *P*=0.2435). In line with previous results, LPA was unable to elicit NLRP3 secretion. Since DG(40:6) was able to induce NLRP3 secretion, Casp1 protein expression in DG(40:6)-stimulated monocytes (Figure 6B) was investigated. Exposure to DG(40:6) resulted in a significant increase in Casp1α (DMSO versus 20 μM, *P*<0.05) and an increasing trend in Casp1β, Casp1γ (DMSO versus 20 μM, *P*<0.1308), as well as Casp1δ (DMSO versus 20 μM, *P*<0.1837; Figure 6C). Similar to Lp(a) stimulation of monocytes, this was accompanied with a dose-dependent increase in IL-1β secretion (Figure 6D), whereas no secretion of IL-18 was detected (data not shown). Together, these results indicate that DGs can activate the NLRP3 inflammasome pathway in monocytes, leading to increased IL-1β secretion. To further investigate this Lp(a)/DG/NLRP3-inflammsome axis, we pretreated monocytes with the NLRP3 inhibitor CRID3 (1 µM) before stimulation with Lp(a). Inhibition of the NLRP3 inflammasome was able to reduce, albeit not significant, Lp(a)-induced IL-1β secretion (Δcytokine secretion, 69.6 pg/mL; 70%; Figure 7A), indicating that NLRP3 inflammasome activation is partially responsible for Lp(a)-induced inflammation. Although Lp(a) induced Casp1 secretion (*P*=0.0168), NLRP3 inhibition did not result in a reduction of Casp1 secretion. NLRP3 inhibition did not lower Lp(a)-induced IL-8 and IL-6 (*P*>0.9999 and *P*>0. 9999, respectively; Figure 7B), suggesting that besides the NLRP3 inflammasome pathway, additional inflammatory pathways are involved in Lp(a)-induced inflammation.

### DGs and LPA Facilitate Monocyte Extravasation

**Figure 8.**
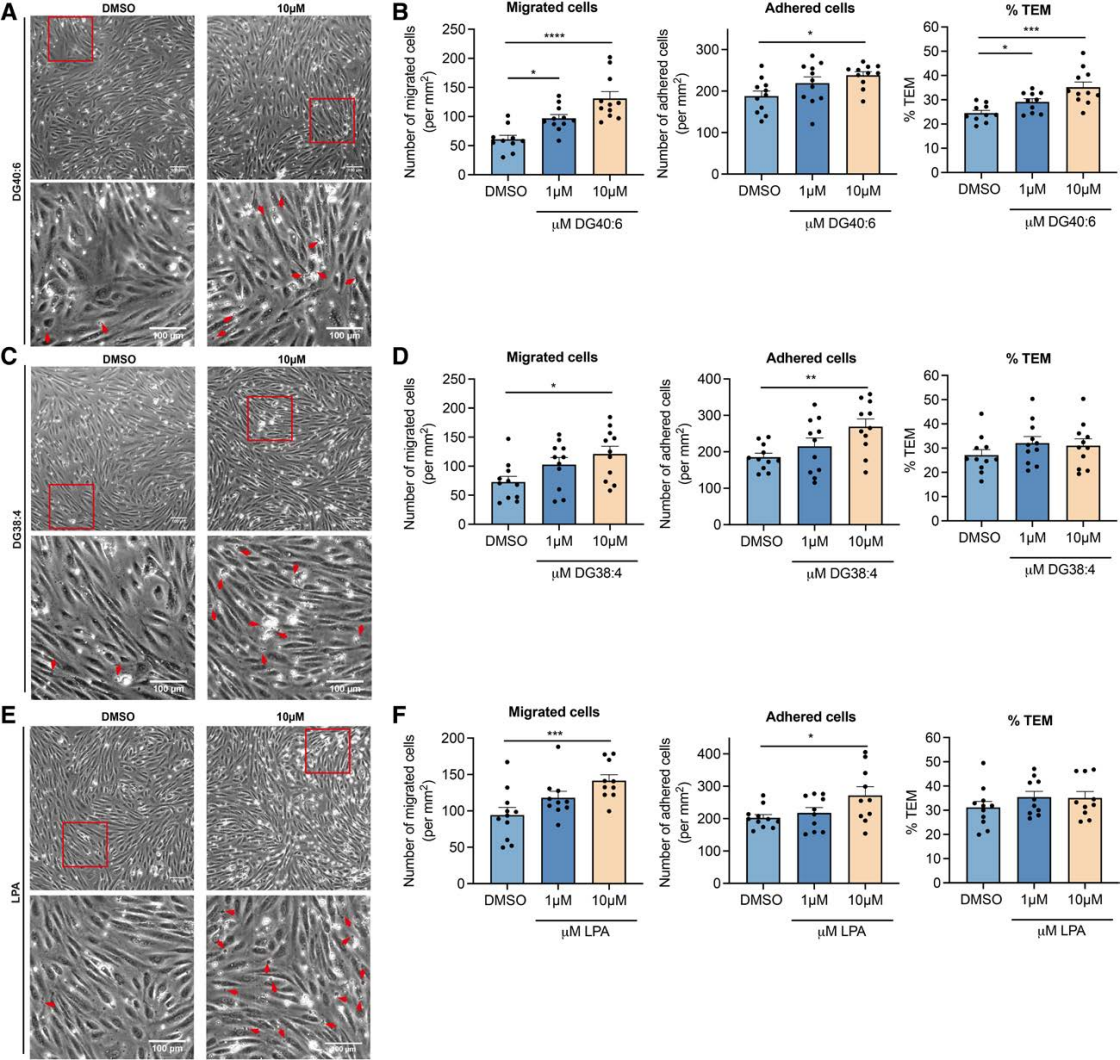
**Diacylglycerols (DGs) and lysophosphatidic acid (LPA) facilitate monocyte extravasation. A**, Representative images of monocyte transendothelial migration (TEM), which were stimulated with either DMSO (dimethyl sulfoxide) as solvent control or 10 μM DG(40:6). Transmigrated monocytes are represented by black cells with a red asterisk and adhered monocytes by white cells. Scale bar, 100 µm. **B**, Quantification of the number of migrated cells, number of adhered cells, and the percentage of migrated cells after stimulation with 1 μM, 10 μM DG(40:6), or DMSO control. **C**, Representative images of TEM of monocytes stimulated with DMSO control or 10 μM DG(38:4). Transmigrated monocytes are represented by black cells with a red asterisk and adhered monocytes by white cells. Scale bar, 100 µm. **D**, Quantification of the number of migrated cells, number of adhered cells, and the percentage of migrated cells after stimulation with 1 μM, 10 μM DG(38:4), or DMSO control. **E**, Representative images of TEM of monocytes stimulated with DMSO control or 10 μM LPA. Transmigrated monocytes are represented by black cells with a red asterisk and adhered monocytes by white cells. Scale bar, 100 µm. **F**, Quantification of the number of migrated cells, number of adhered cells, and the percentage of migrated cells after stimulation with 1 μM, 10 μM LPA, or DMSO control. All data are mean±SEM. Data represent 4 independent experiments. Grubbs outlier tests were performed to identify outliers. This was followed by a Shapiro-Wilk test to test for normality. When data followed a normal distribution, an ordinary 1-way ANOVA was performed followed by Dunnett multiple comparisons test. When the data were not normally distributed, a Kruskal-Wallis test was performed followed by a Dunn multiple comparisons test. *P<0.05, **P<0.01, ***P<0.001, ****P<0.0001.

Monocyte recruitment and extravasation through the endothelium is a major driver of atherogenesis and one of the initial steps in atherosclerosis. To assess whether this DG-induced increase in monocyte activation leads to changes in their adhesive and migratory capacity, we performed monocyte transendothelial migration assays with DG- and LPA-stimulated monocytes (Figure 8). Healthy human monocytes were stimulated with either 1 or 10 μM DG(40:6), DG(38:4), or LPA and added to a TNFα-stimulated HAEC monolayer. The number of adhered monocytes increased significantly after incubation with 10 μM DG(40:6; *P*=0.0127; Figure 8A and 8B), DG(38:4; *P*=0.0072; Figure 8C and 8D), and LPA (*P*=0.0247; Figure 8E and 8F). Similarly, the number of migrated monocytes was increased after stimulation with 1 μM (*P*=0.0356) and 10 μM DG(40:6; *P*<0.0001; Figure 8B), DG(38:4; *P*=0.0132; Figure 8D), and LPA (*P*=0.0009; Figure 8F). An increase in the percentage of migrated monocytes was observed after exposure to 10 μM DG(40:6; *P*=0.0002; Figure 8B). Collectively, these findings suggest that, in addition to augmenting cytokine secretion and gene expression, DGs and LPA enhance monocyte extravasation, thereby contributing to the advancement of atherosclerosis.

### Lp(a) Colocalizes With M1 Macrophages in Atherosclerotic Lesions

**Figure 9.**
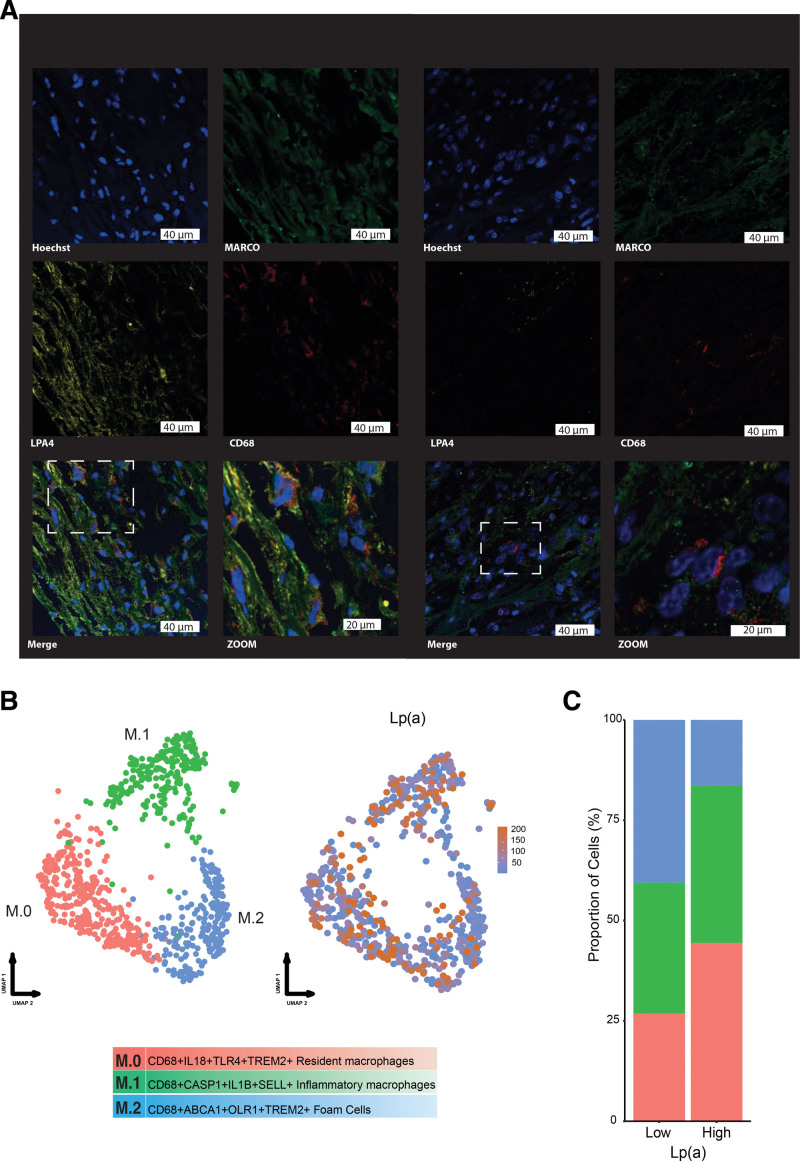
**Lipoprotein(a) (Lp[a]) colocalizes with M1 macrophages in atherosclerotic lesions. A**, Images representing human carotid plaques derived from patients with high Lp(a) and low Lp(a) levels. Nuclei were stained with Hoechst (blue). Macrophages were stained with the pan-macrophage marker CD68 (red). M1 macrophages were determined by colocalization of CD68 and MARCO (macrophage receptor with collagenous structure; green). Apo(a) was stained with LPA4 (yellow). Scale bars, 40 and 20 µm for the zoom images. **B**, tSNE visualization of 3 distinct macrophage populations. **C**, Bar plot demonstrating the distribution of macrophage clusters in the Lp(a) high and low groups. ABCA1 indicates ATP-binding cassette transporter 1; Casp1, caspase 1; IL, interleukin; OLR1, oxidized low-density lipoprotein receptor 1; SELL, selectin L; TLR4, toll-like receptor 4; TREM2, triggering receptor expressed on myeloid cells 2; and tSNE, t-distributed stochastic neighbor embedding.

After demonstrating in vitro that Lp(a)-associated lipids can induce a proinflammatory response in monocytes, we investigated whether the accumulation of Lp(a) coincided with proinflammatory macrophages in human atherosclerotic lesions. Therefore, we stained for apo(a) (LPA4), the pan-macrophage marker CD68, and the M1 macrophage marker MARCO in atherosclerotic lesions from patients with high Lp(a) levels included in the Athero-Express Biobank (UMC Utrecht; Figure 9). Confocal microscopy imaging of human plaques demonstrated increased colocalization of CD68 in patients with high Lp(a) levels compared with patients with low Lp(a) levels, indicating an increase in M1 macrophages. To confirm the specificity of the LPA4 antibody, frozen healthy aorta was stained with hematoxylin and LPA4 (apo[a]). We demonstrate minimum LPA4 staining, indicating no nonspecific binding of the antibody (Figure S7A). In addition, isotype controls were used as a negative control (Figure S7B). For further investigating the phenotype of these plaque macrophages, scRNA-seq was performed for the macrophages in the plaques of patients with high (≥75 nmol/L, ≥30 mg/dL; n=10) and low (≤5.7 nmol/L, ≤2.28 mg/dL; n=10) Lp(a) (Figure 9B). The plaques from Lp(a)-high patients demonstrated an enrichment of the CD68^+^IL-18^+^TLR4^+^ (toll-like receptor) TREM2^+^ (triggering receptor expressed on myeloid cells) resident macrophages and CD68^+^CASP1^+^IL-1B^+^SELL^+^ (selectin L) inflammatory macrophages compared with Lp(a)-low patients (Figure 9C). Targeted analysis showed expression of genes involved in the NLRP3 inflammasome pathway (*IL1B* and *CAPS1*), leukocyte migration (*CCL4*, *CCL3*, and *CXCL8*), and NF-κB signaling (*NFKB1*, *TNF*, and *TNFAIP3*; Figure S8). Overall, these data are in line with our in vitro findings that Lp(a)-associated lipids induce the polarization of monocytes toward a proinflammatory phenotype, which, in part, is NLRP3 inflammasome mediated.

### Lp(a) Lowering Had No Effect on DGs as a Lipid Class As Well As Inflammasome Markers

**Figure 10.**
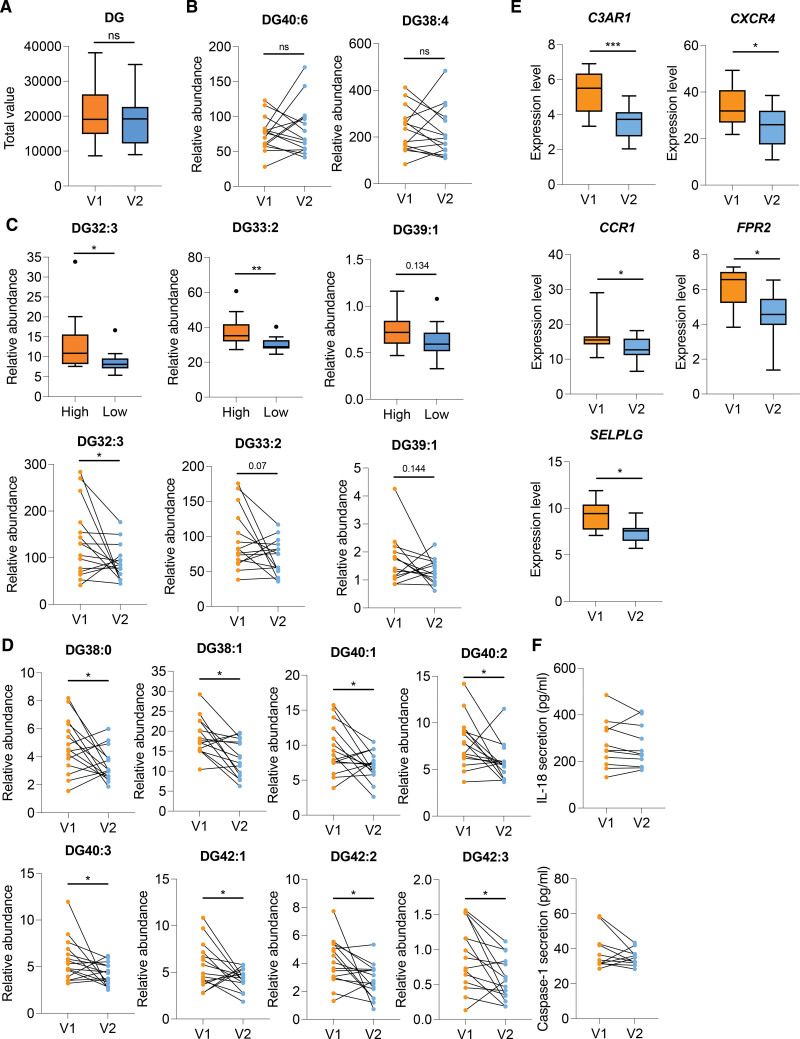
**Lipoprotein(a) (Lp[a]) lowering had no effect on diacylglycerols (DGs) as a lipid class, as well as inflammasome markers. A**, Boxplots demonstrating the total value of DG after potent Lp(a) lowering by apo(a) antisense (pelacarsen). Total value is the sum of the relative abundance of all identified lipid species. For the lipidomics analysis, a Student *t* test with post hoc Bonferroni correction was used for statistical comparison between the 2 groups. **B**, Graphs demonstrating the relative abundance of DG(40:6) and DG(38:4) after potent Lp(a) lowering by apo(a) antisense (pelacarsen). **C**, Boxplots and graphs demonstrating the relative abundance of DG(32:3), DG(33:2), and DG(39:1) between healthy individuals with high and low levels of Lp(a), as well as after potent Lp(a) lowering by apo(a) antisense (pelacarsen) in patients with cardiovascular disease. **D**, Graphs demonstrating the relative abundance of DG(38:0), DG(38:1), DG(40:1), DG(40:2), DG(40:3), DG(42:1), DG(42:2), and DG(42:3) after potent Lp(a) lowering by apo(a) antisense (pelacarsen). **E**, Box plots of differentially expressed chemotaxis- related genes before and after pelacarsen. *P*-adjusted values <0.1 were considered statistically significant. **F**, IL (interleukin)-18 and Casp1 (caspase-1) secretion in plasma before and after pelacarsen treatment. Data were analyzed using a 2-tailed paired *t* test. *P<0.05, **P<0.01, ***P<0.001.

To assess whether potent Lp(a) lowering by apo(a) antisense (pelacarsen) can decrease DG and LPA plasma levels, we conducted complete plasma lipidomics analysis before (V1) and after (V2) 26 weeks of pelacarsen treatment. The cohort included 14 patients with CVD with elevated Lp(a) levels (median, 82 mg/dL [205 nmol/L]) and was described previously by Stiekemaet al.^14^ Lp(a) lowering (mean absolute change, −50.6 [52.6] mg/dL) did not result in a decrease in the DG lipid class as a whole (Figure 10A). We further investigated the effect of Lp(a) lowering on individual DG lipid species. Whereas Lp(a) lowering did not result in a decrease in DG(40:6) and DG(38:4; Figure 10B), the lipid species DG(32:3), DG(33:2), and DG(39:1), which were also upregulated in individuals with high levels of Lp(a) compared with low (Figure 10C), did decrease albeit not significantly for all after pelacarsen treatment (*P*=0.0497, *P*=0.0703, and *P*=0.1441, respectively). Interestingly, this decrease in DG(32:3) and DG(33:2) showed a trend with the decrease in OxPL-apo(a) after pelacarsen treatment (*P*=0.0955 and *P*=0.1041; Figure S9). Additionally, DG(38:0), DG(38:1), DG(40:1), DG(40:2), DG(40:3), DG(42:1), DG(42:2), and DG(42:3) were decreased after Lp(a) lowering (Figure 10D). Furthermore, a decrease in the chemotaxis-related genes *C3AR1* (*P*<0.001), *CXCR4* (*P*=0.033), *CCR1* (*P*=0.033), *FPR2* (*P*=0.037), and *SELPLG* (*P*=0.041) was observed in the circulating monocytes of these patients (Figure 10E). However, IL-18 and Casp1 secretion in plasma did not decrease significantly after Lp(a) lowering (Figure 10F). IL-1β was not able to be detected in plasma (data not shown). Overall, lowering Lp(a) levels using pelacarsen therapy can diminish certain circulating DG lipid species in patients with CVD with elevated Lp(a) but not the whole lipid class of DGs.

## DISCUSSION

In the current study, our findings demonstrate that while the inhibition of OxPL substantially decreases monocyte activation, a persistent IL-8–mediated residual inflammation remains. Furthermore, we observed no reduction in IL-1β levels after OxPL inhibition. These findings strongly suggest that there are additional Lp(a)-derived proinflammatory mediators that contribute to Lp(a)-induced monocyte inflammation. This is now supported by extensive lipidomics analysis, showing that individuals with elevated Lp(a) levels have a distinct lipidome compared with healthy individuals with low/normal Lp(a) levels. This altered Lp(a) lipidome is characterized by an increased abundance of DGs and LPA. DGs and the LPA precursor LPC are enriched in the Lp(a) fraction, indicating that DGs and LPC are Lp(a)-associated lipid species.

Functional assessment of DG(40:6), DG(38:4), and LPA(18:0) found in individuals with elevated Lp(a) levels demonstrated a dose-dependent increase in the secretion of proinflammatory cytokines IL-6, IL-8, and IL-1β after DG stimulation of healthy human monocytes. In addition, LPA stimulation did not affect monocyte activation, as attested by no observed changes in cytokine secretion. Mechanistically, this DG-induced IL-1β secretion was partly mediated via the NLRP3 inflammasome pathway. Furthermore, the activation of monocytes by DGs and LPA has been shown to enhance their transendothelial migratory capacity. This finding is consistent with previous studies,^4^ which have demonstrated that monocytes derived from individuals with elevated Lp(a) levels exhibit an augmented migratory phenotype ex vivo. This phenotype is characterized by increased transendothelial migration and elevated expression of chemokine, adhesion, and transmigration surface markers.^4^ Finally, it was observed that plasma levels of DGs (as a major lipid class), as well as Casp1 and IL-18, were not influenced by potent Lp(a)-lowering therapy using pelacarsen in patients with CVD with elevated Lp(a) levels. Overall, the present study shows additional Lp(a)-derived proinflammatory mediators that play a role in Lp(a)-induced monocyte inflammation.

### Potential Origin of DG and LPA

DGs have been shown to be intermediates of lipid metabolism and can be formed via multiple pathways.^32^ Previous research has shown that Lp(a) is enriched with the enzymes Lp-PLA2 (lipoprotein-associated phospholipase A2) and ATX, which play a role in lipid metabolism.^10,11,31,33^ Lp-PLA2 is enriched in both mass and activity on Lp(a) and mediates the hydrolysis of OxPL, leading to the generation of LPC.^33^ LPC can be converted into LPA by ATX.^10,11,31^ LPA has multiple degradation pathways: (1) production of glycerol 3-phosphate by LPL (lysophospholipase), (2) conversion into PA by LPA acyltransferase, which could lead to the generation of DG via PAP (phosphatidate phosphatase), (3) generation of DG by LLP1 (lipid phosphate phosphatase 1), and (4) conversion in monoacylglycerol by LPP1 or PAP, which could lead to the formation of DG via MGAT (monoacylglycerol acyltransferase).^26,34^ Combined, these studies and the positive association between OxPL-apo(a), DG(32:3), and DG(33:2; Figure S6) suggest a possibility of a lipid metabolic pathway consisting of the following route: OxPL→PLC→lysoPC→lysoPA→PA or monoacylglycerol→DG. It would be of interest to investigate whether Lp(a) is enriched with the enzymes crucial for the downstream steps, such as LPA acyltransferase, PAP, and LLP1.

### Potential Atherogenic Role of DGs

Increased DG levels have been linked to cardiovascular risk previously.^28,35^ Lee et al^28^ demonstrated that LDL from patients with acute coronary syndrome had a 3.5-fold increase in (18:0, 22:6)-DG compared with patients with stable coronary artery disease, indicating a possible role for DGs in active disease. We now demonstrated that DGs and Lp(a) induce IL-1β secretion, which is partly driven by NLRP3 inflammasome activation. This NLRP3 inflammasome pathway has been shown to contribute to vascular inflammation and atherosclerosis progression.^36,37^ Moreover, the CANTOS trial (Canakinumab Antiinflammatory Thrombosis Outcome Study) using the monoclonal antibody canakinumab targeting IL-1β and the LoDoCo2 trial (Low-Dose Colchicine) using low-dose colchicine demonstrated that targeting aspects of the NLRP3 inflammasome pathway lowered cardiovascular events independent of lipid-lowering therapy.^38–40^ Our findings that elevated Lp(a)-associated lipids activate the NLRP3 inflammasome might indicate that patients with high Lp(a) levels could also benefit from therapy targeting the inflammasome.

Matrix-assisted laser desorption/ionization mass spectrometry imaging of human carotid atherosclerotic plaques demonstrated that DGs accumulate in areas with the thrombus-associated protein fibrin, pointing to a role of DGs in atherothrombosis.^41^ This is in line with our finding that elevated Lp(a) levels (>137 nmol/L; >80th cohort percentile) have been associated with an increased risk of 30-day major adverse cardiovascular events, mainly stroke, following carotid endarterectomy.^42^ With the recent advancements in single-cell technologies, it would be of interest to combine matrix-assisted laser desorption/ionization mass spectrometry imaging with spatial transcriptomics. This would provide us with the opportunity to explore the spatial distribution of DGs and LPA, as well as provide mechanistic insight into how these lipids contribute to the modulation of the immune cell phenotype in atherosclerotic lesions. While there is evidence suggesting a potential association between Lp(a)-associated DGs and the inflammatory phenotype of monocytes, further research is needed to fully understand the complex mechanisms involved and to establish a definitive causal relationship.

### LPC and LPA and Their Association With Proinflammatory Response

The conversion of LPC into LPA by Lp(a)-associated ATX is in line with the increased abundance of LPA in the plasma of healthy individuals with elevated Lp(a) levels. Although exposure to LPA did not elicit an inflammatory response in monocytes and HAECs, LPA stimulation did increase the migration capacity of monocytes. Other studies also demonstrated that LPA induces monocyte chemotaxis via LPA receptor 2 and actin cytoskeletal mobilization, independent from cytokine secretion. Because we treated monocytes with LPA for 18 hours before assessing their migratory capacity, it is tempting to speculate that LPA receptor 2 could have been activated and actin cytoskeleton remodeling could have taken place, thereby facilitating monocyte migration via this alternative pathway.^43,44^

### Potent Lp(a) Lowering and Lipidome Changes

We demonstrated that potent Lp(a) lowering by pelacarsen therapy decreases certain DG lipid species (DG(32:3), DG(33:2), DG(39:1), DG(38:0), DG(38:1), DG(40:1), DG(40:2), DG(40:3), DG(42:1), DG(42:2), and DG(42:3)) in patients with CVD with elevated Lp(a) levels. This, however, did not affect DGs as a major lipid class. Previously, it has been shown that pelacarsen resulted in a 67% to 72% reduction of Lp(a), which was accompanied with a 27% to 37% decrease in OxPL-apo(a).^45^ Although Lp(a) is the preferential carrier of OxPLs in plasma, the disparity between the percentage of decrease in Lp(a) and OxPL-apo(a) could be explained by the ability of OxPLs to bind other lipoproteins, such as oxidized LDL.^7,46^ Since previous studies have shown that DGs can be carried by other lipoproteins such as VLDL (very-low-density lipoprotein), LDL, and HDL,^27,47^ it is possible that DGs are also associated to other lipoproteins, thereby providing a rationale to why the majority of the DG lipid species do not decrease after pelacarsen therapy.

The decrease in chemotaxis-related genes (*C3AR1*, *CXCR4*, *CCR1*, *FBR2*, and *SELPLG*) in monocytes of pelacarsen-treated patients was in line with previous research demonstrating that lowering of Lp(a) and its associated OxPLs resulted in the reduction of the proinflammatory phenotype of circulating monocytes, as well as a decrease in monocyte transendothelial migration capacity.^14^ Since there is a profound reduction in OxPLs, we cannot conclude that this decrease in chemotaxis-related genes is due to a reduction in certain Lp(a)-associated DGs. As we did not observe major differences in NLRP3 activation before and after pelacarsen therapy, it could be that with DGs associating with other lipoproteins, the NLRP3 inflammasome pathway can still be activated, explaining the lack of reduction in IL-18 and Casp1 after pelacarsen therapy.

### Conclusions

The present study shows a potential role of Lp(a)-associated DGs and LPA in Lp(a)-induced monocyte inflammation.

## ARTICLE INFORMATION

### Acknowledgments

The authors thank Dr Laura Bosmans, Hans Jansen, and Alinda Schimmel for their excellent technical support throughout this study and Xiang Zhang for the lively discussions. The graphic abstract in this article was created with BioRender.com.

### Sources of Funding

This work was supported by the Netherlands Organization for Scientific Research. J. Kroon received a VENI grant from ZonMW (91619098) and was supported by the Dutch Heart Foundation (Senior Scientist Dekker grant 03-004-2021-T045). This work was funded by the European Union (European Research Council (ERC) starting grant, ENDOMET-STEER, 101076407). The views and opinions expressed are, however, those of the authors only and do not necessarily reflect those of the European Union or the European Research Council Executive Agency. Neither the European Union nor the granting authority can be held responsible for them. J. Kroon received a preclinical research grant from Oxitope Pharma B.V. S. Tsimikas was supported by National Institutes of Health (NIH) R01 HL159156. The Pelacarsen studies were funded by Ionis Pharmaceuticals.

### Disclosures

S. Tsimikas is a coinventor and receives royalties from patents owned by the University of California San Diego (UCSD), is a cofounder and has an equity interest in Oxitope Pharma and Kleanthi Diagnostics, and has a dual appointment at UCSD and Ionis Pharmaceuticals. The other authors report no conflicts.

### Supplemental Material

Figures S1–S9

Major Resources Table

Tables S1–S4

Data Sets S1–S3

Full Unedited Blots for Figure 6

R Script S1

## Supplementary Material


