# Supplementary figures and images for: Diacylglycerols and Lysophosphatidic Acid, Enriched on Lipoprotein(a), Contribute to Monocyte Inflammation
Source: Arterioscler Thromb Vasc Biol. 2024 Jan 25;44(3):720–40. doi: 10.1161/ATVBAHA.123.319937 (PMC10880937; doi:10.1161/ATVBAHA.123.319937)

**A. Caspase-1 Set 1: Representative blot**

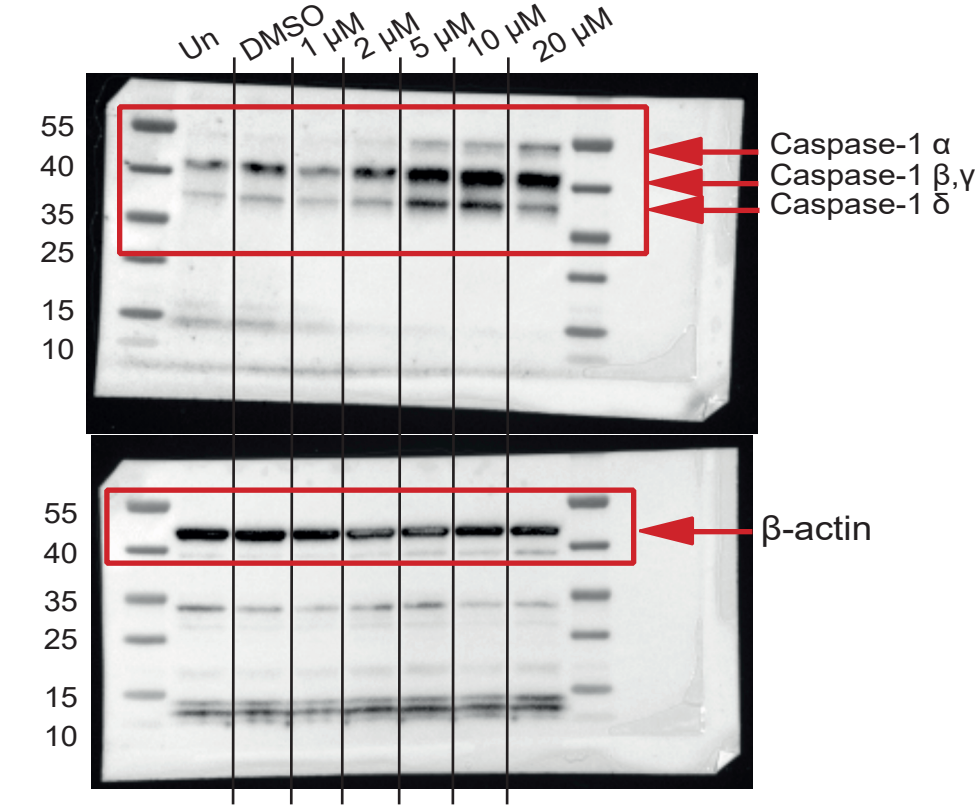

**B. Caspase-1 Set 2**

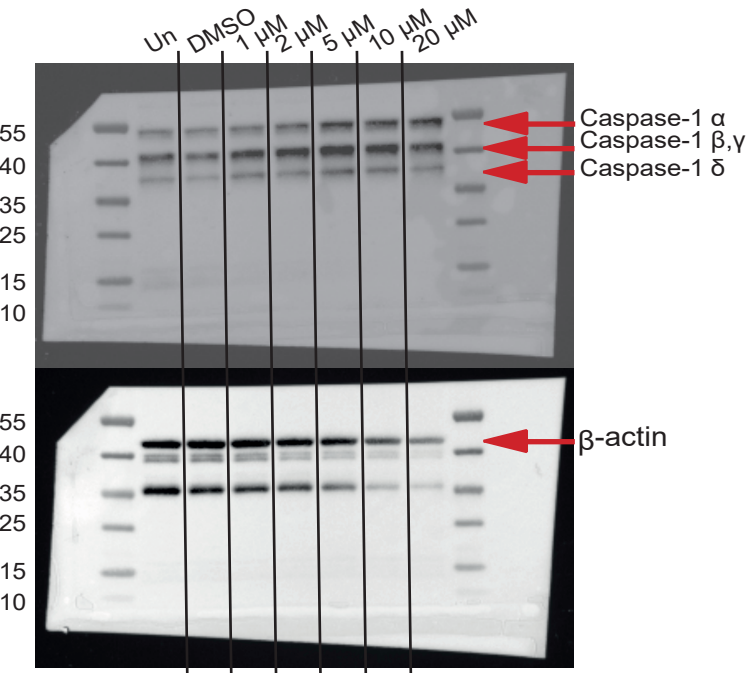

**C. Caspase-1 Set 3**

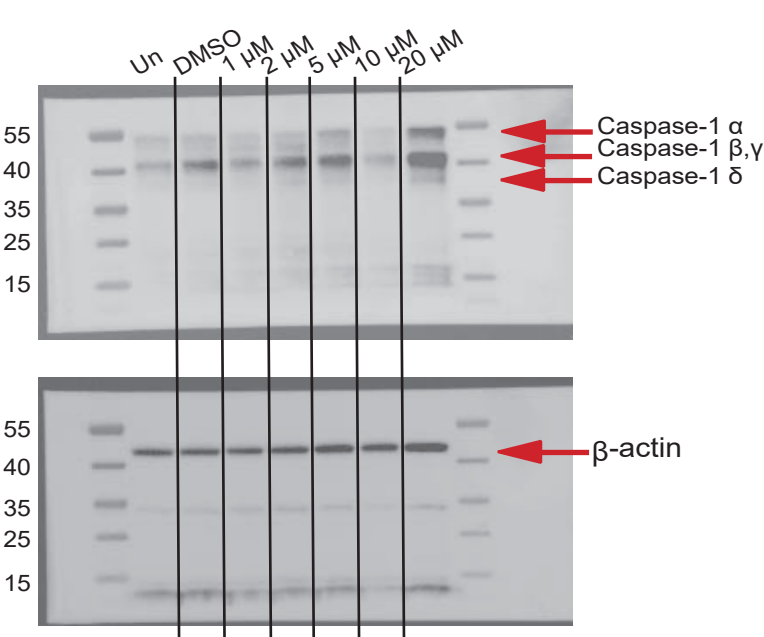

Supplement: Supplementary file 5 [file atv-44-720-s005.pdf]
