# Supplementary material for: Diacylglycerols and Lysophosphatidic Acid, Enriched on Lipoprotein(a), Contribute to Monocyte Inflammation
Source: Arterioscler Thromb Vasc Biol. 2024 Jan 25;44(3):720–40. doi: 10.1161/ATVBAHA.123.319937 (PMC10880937; doi:10.1161/ATVBAHA.123.319937)
Supplement: Supplementary file 6 [file atv-44-720-s006.pdf]

SUPPLEMENTAL MATERIALS

Diacylglycerols and lysophosphatidic acid, enriched on Lipoprotein(a), contribute to monocyte inflammation

Kim E. Dzobo[1,2], Arjen Cupido[3*], Barend M. Mol[4*,] Lotte C.A. Stiekema[3], Miranda Versloot[1,2,] Maaike Winkelmeijer[1], Jorge Peter[1], Anne-Marije Pennekamp[1], Stefan Havik[1], Frédéric M. Vaz[5], Michel van Weeghel[5], Koen H.M. Prange[6], Johannes H.M. Levels[1], Menno PJ de Winther[6], Sotirios Tsimikas[7], Albert K. Groen[1], Erik S.G. Stroes[3], Dominique P.V. de Kleijn[4] and Jeffrey Kroon[1,2,8,9]

* Shared authors

1. Amsterdam UMC location University of Amsterdam, Department of Experimental Vascular Medicine, Amsterdam Cardiovascular Sciences, Meibergdreef 9, Amsterdam, Netherlands.
2. Amsterdam Cardiovascular Sciences, Atherosclerosis & Ischemic Syndromes, Amsterdam, The Netherlands
3. Amsterdam UMC location University of Amsterdam, Department of Vascular Medicine, Amsterdam Cardiovascular Sciences, Meibergdreef 9, Amsterdam, Netherlands
4. Department of Vascular Surgery, University Medical Centre Utrecht, Utrecht, The Netherlands.
5. Amsterdam UMC location University of Amsterdam, Core Facility Metabolomics, Meibergdreef 9, 1105 AZ Amsterdam, The Netherlands.
6. Amsterdam UMC location University of Amsterdam, Department of Medical Biochemistry, Amsterdam Infection and Immunity, Meibergdreef 9, Amsterdam, Netherlands.
7. Division of Cardiovascular Medicine, Sulpizio Cardiovascular Center, University of California San Diego, La Jolla, California.
8. Laboratory of Angiogenesis and Vascular Metabolism, VIB-KU Leuven Center for Cancer Biology, VIB, 3000 Leuven, Belgium
9. Laboratory of Angiogenesis and Vascular Metabolism, Department of Oncology, KU Leuven and Leuven Cancer Institute (LKI), 3000 Leuven, Belgium

* Address for correspondence: J. Kroon, Ph.D. Amsterdam UMC, location University of Amsterdam, Department of Experimental Vascular Medicine, Room G1.142, Amsterdam Cardiovascular Sciences, Meibergdreef 9, Amsterdam, Netherlands. Phone: +31205661150. Email: j.kroon@amsterdamumc.nl

Supplemental Fig. 1

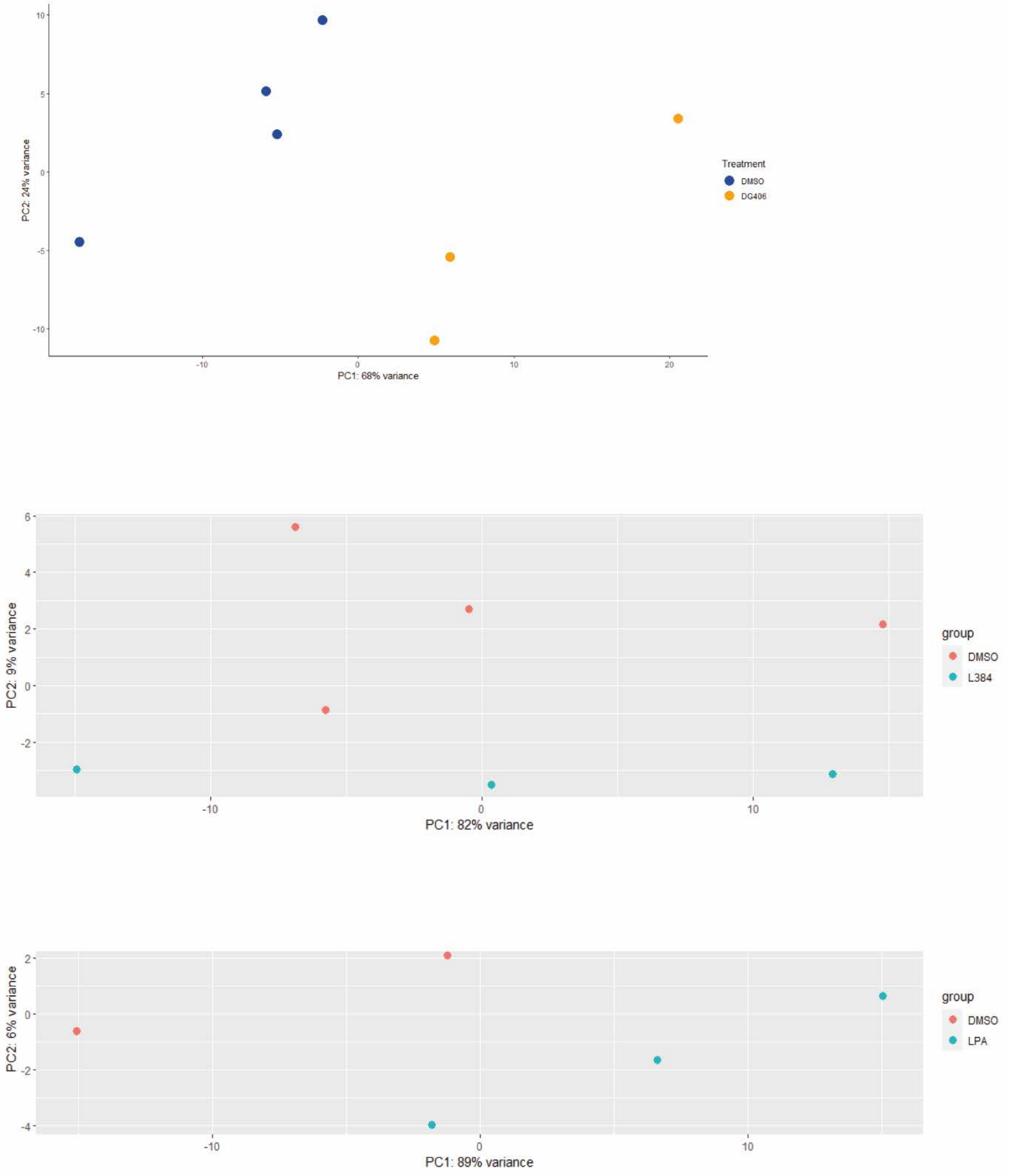

Figure S1: Principal component analysis for DMSO vs 10 µM DG(40:6), DG(38:4) and LPA.

Supplemental Fig. 2

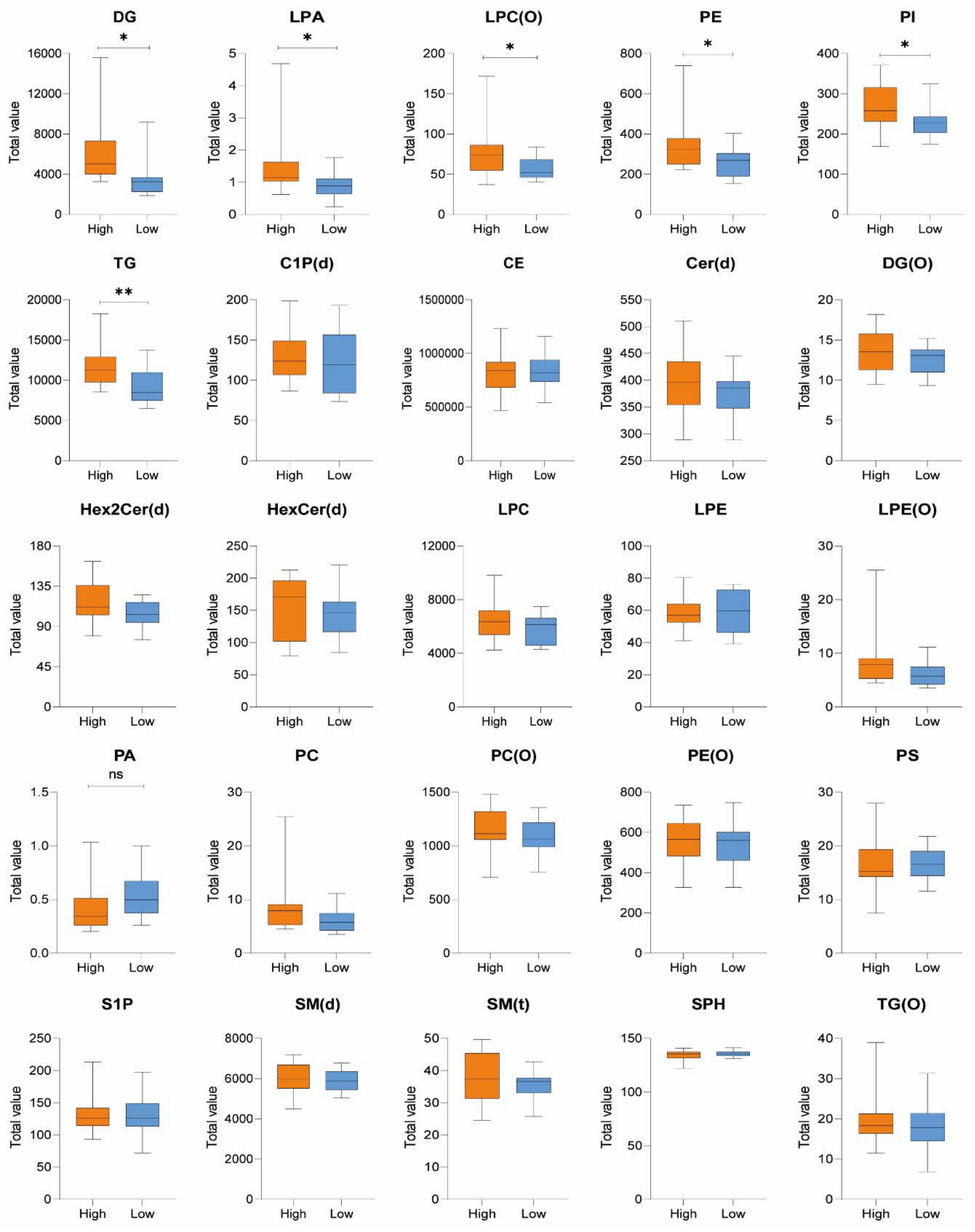

Figure S2: Boxplots demonstrating the relative abundance of 25 major lipid classes in healthy individuals with high levels of Lp(a) (orange) and low Lp(a) levels (blue). A Students *t*-test with post hoc Bonferroni correction was used for statistical comparison between the two groups. **P*<0.05, ***P*<0.01

Supplemental Fig. 3

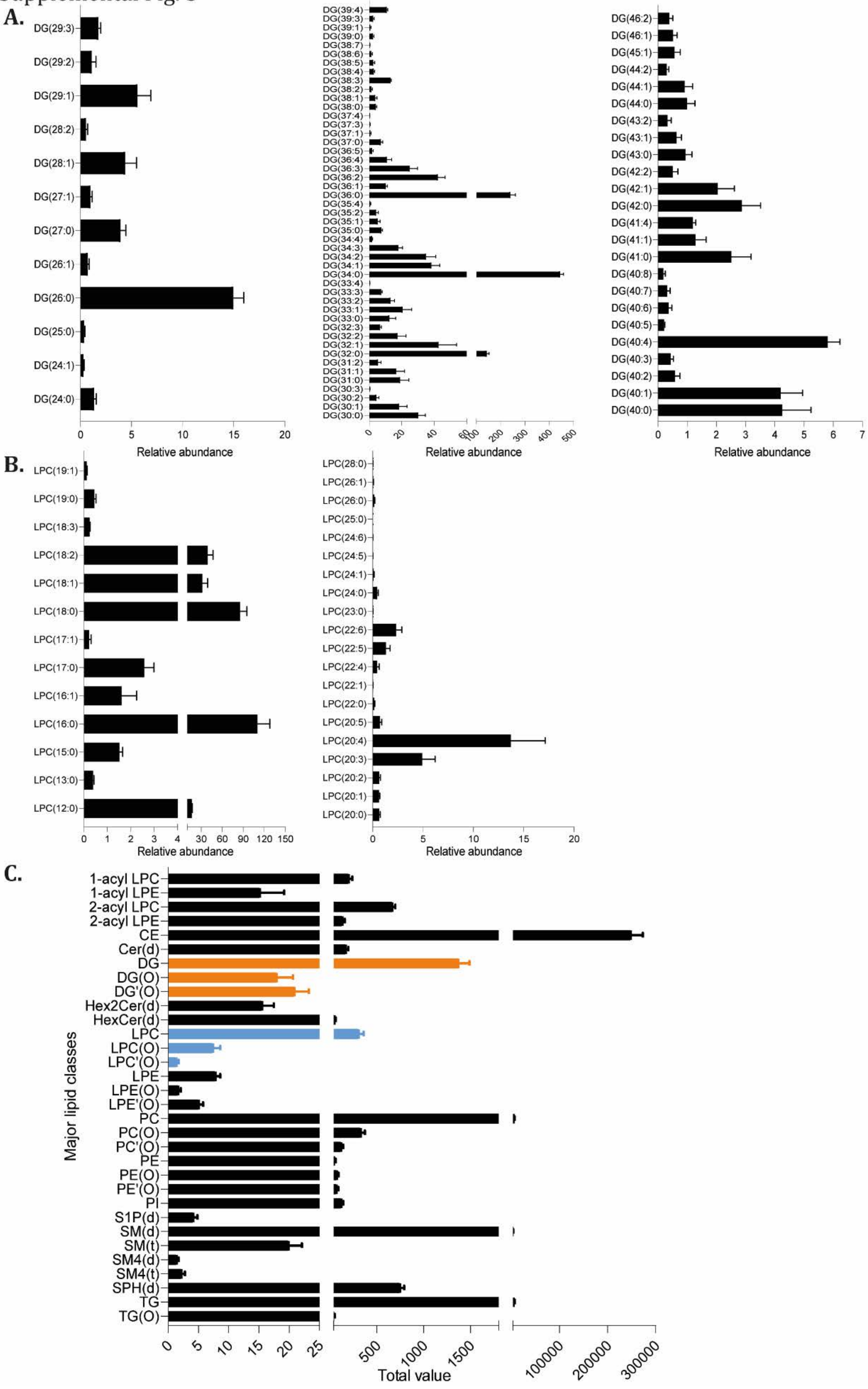

Figure S3: Bar graphs demonstrating **(A)** the individual DG species, **(B)** individual LPC species and **(C)** major lipid classes enriched on Lp(a). With DG, DG(O), DG'(O) highlighted in orange and LPC, LPC(O) and LPC'(O) highlighted in blue.

Supplemental Fig. 4

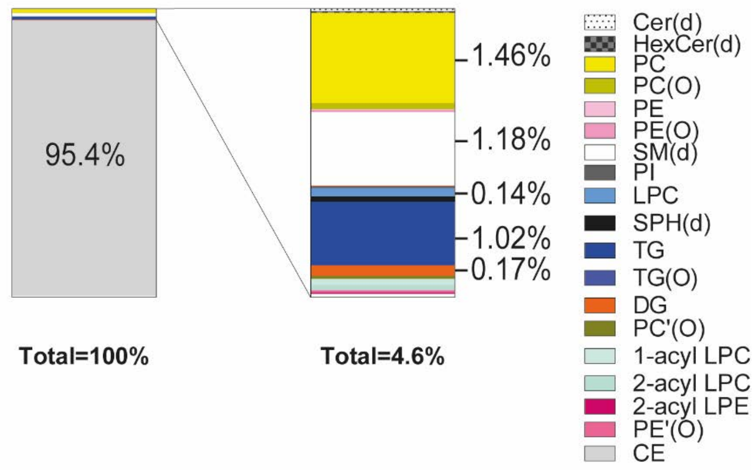

Figure S4: Per major lipid class the mean percentage (%) in the total lipidome of the LDL fraction

Supplemental Fig. 5

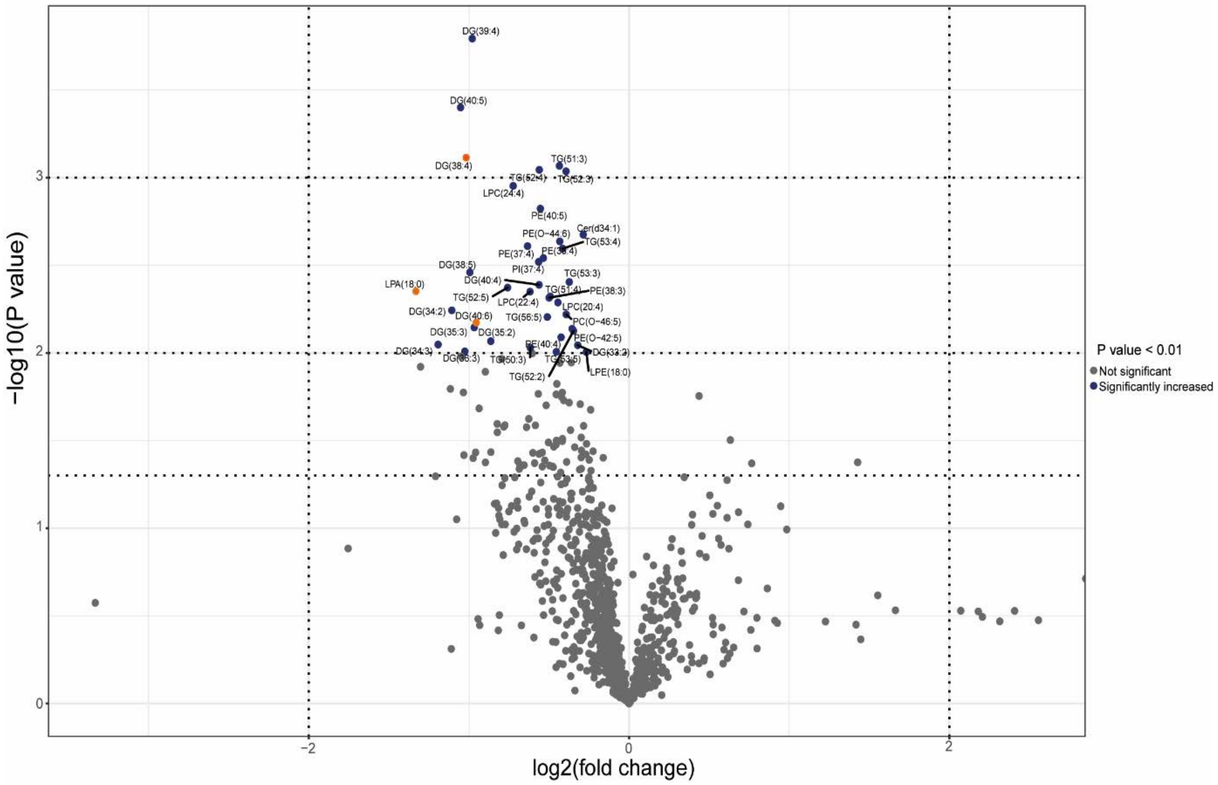

Figure S5: Volcano plot showing 50 most changed lipid species in healthy individuals with high Lp(a) levels compared to healthy individuals with low Lp(a) levels based on p-value. A Students *t*-test with post hoc Bonferroni correction was used for statistical comparison between the two groups. The three horizontal dotted lines indicate p-values of 0.05, 0.01 and 0.001. The two vertical dotted lines indicate log2(fold change) of -2 and 2.

Supplemental Fig. 6

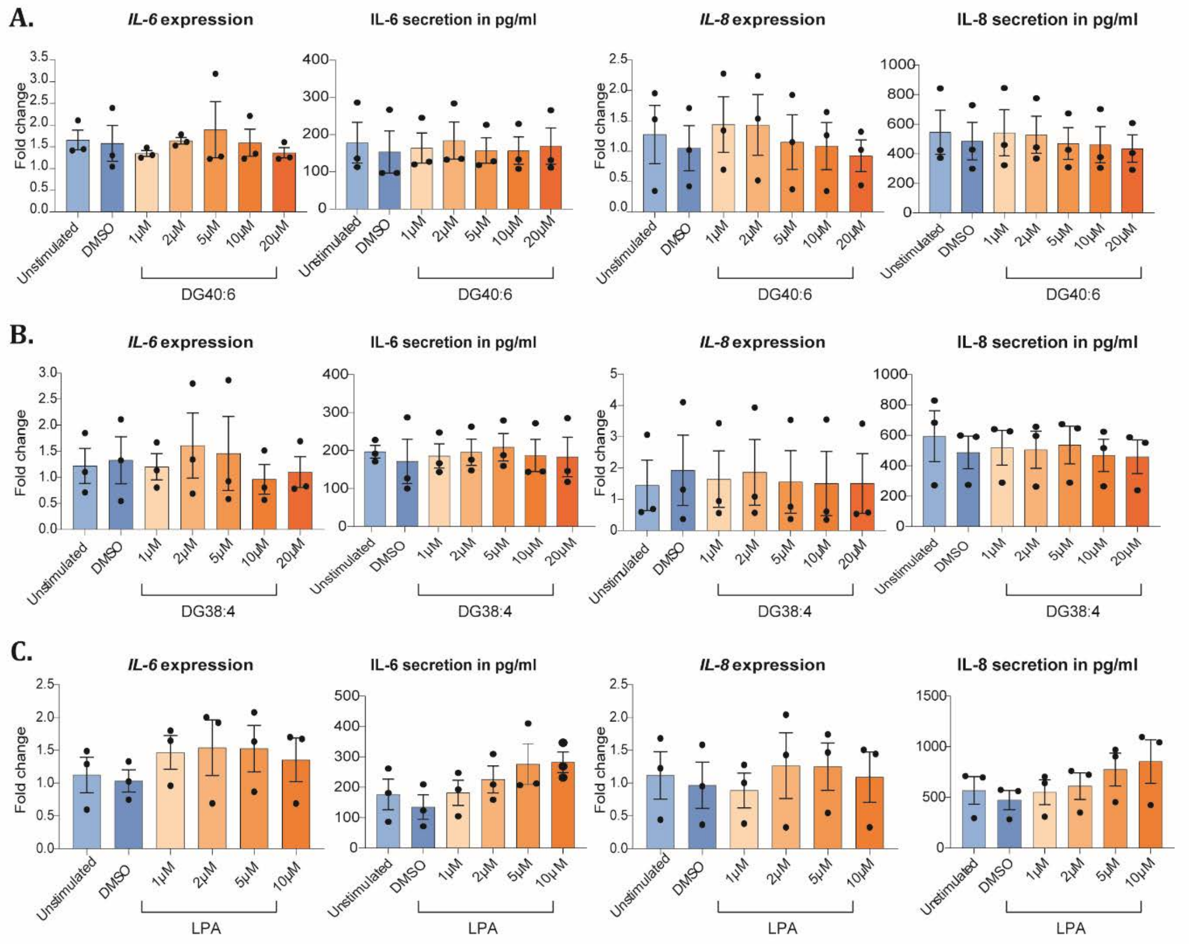

Figure S6: Diacylglycerols and lysophosphatidic acid did not trigger a a pro-inflammatory response in endothelial cells. (A) IL-6 and IL-8 relative expression in HAECs stimulated with increasing concentration of DG(40:6), **(C)** DG(38:4) and **(E)** LPA (18hr stimulation; n=3). **(B)** IL-6 and IL-8 secretion in cell medium of HAECs stimulated with increasing concentration of DG(40:6), **(D)** DG(38:4) and **(F)** LPA (18hr stimulation; n=3).

Supplemental Fig. 7

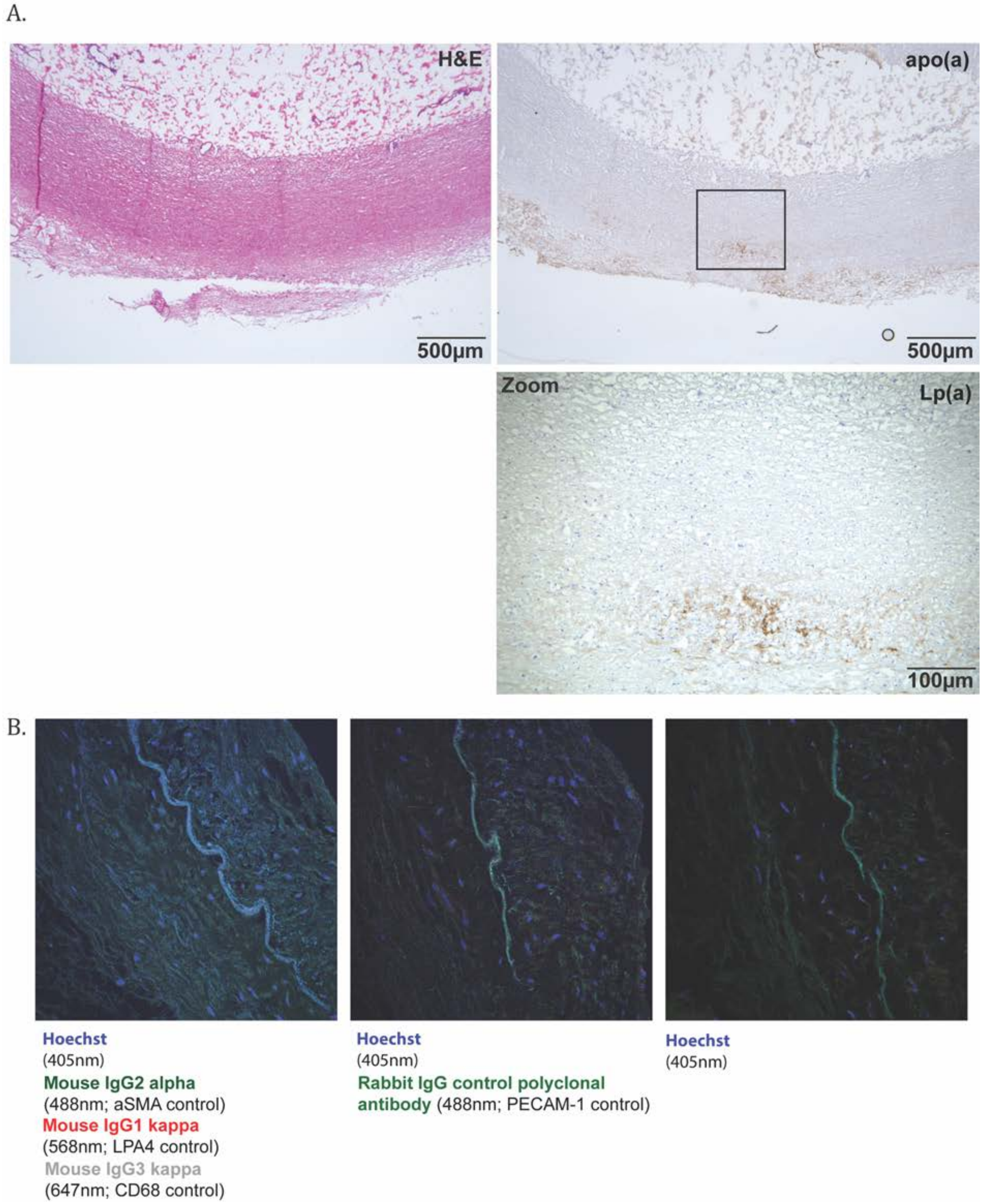

Figure S7: (A) H&E staining and LPA4 staining on healthy human aorta. (B) Isotype controls as a negative control in human atherosclerotic plaques.

Supplemental Fig 8.

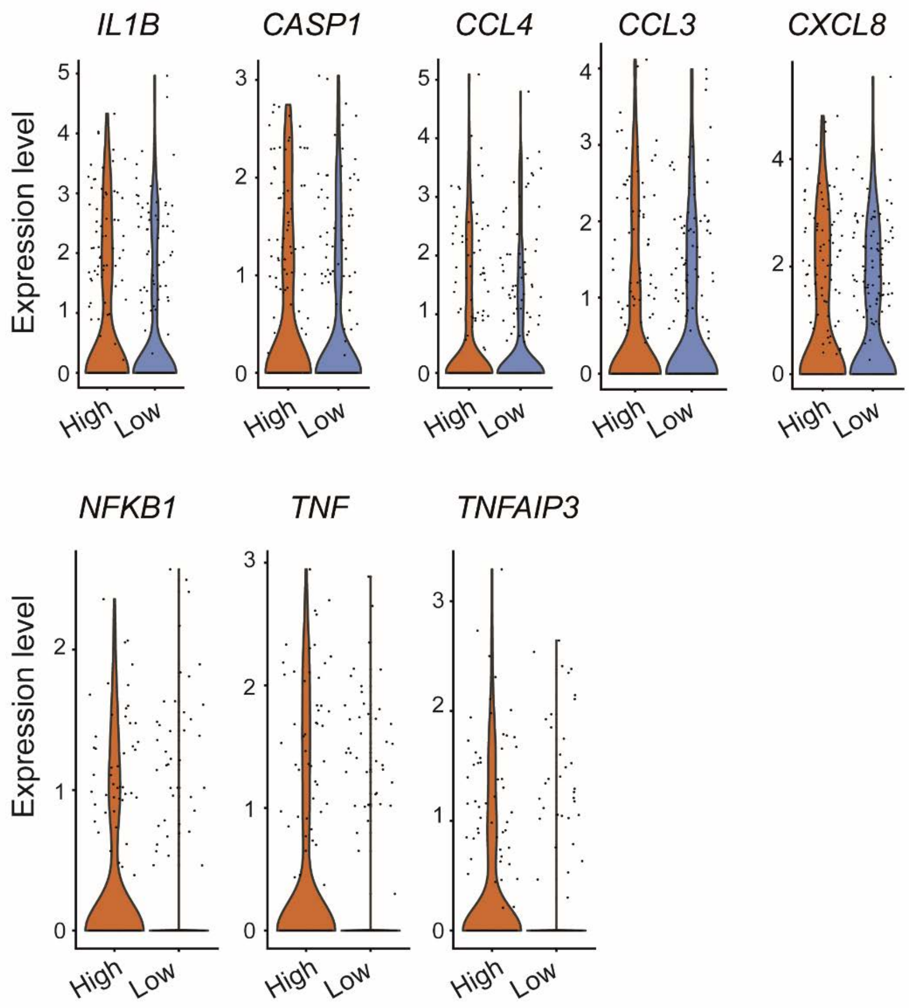

Figure S8: Violin plot displaying the expression of genes involved in the NLRP3 inflammasome pathway (*IL1B*, *CAPS1),* leukocyte migration *(CCL4, CCL3, CXCL8)* and NF-κB signalling *(NFKB1, TNF* and *TNFAIP3)*

Supplemental Fig. 9

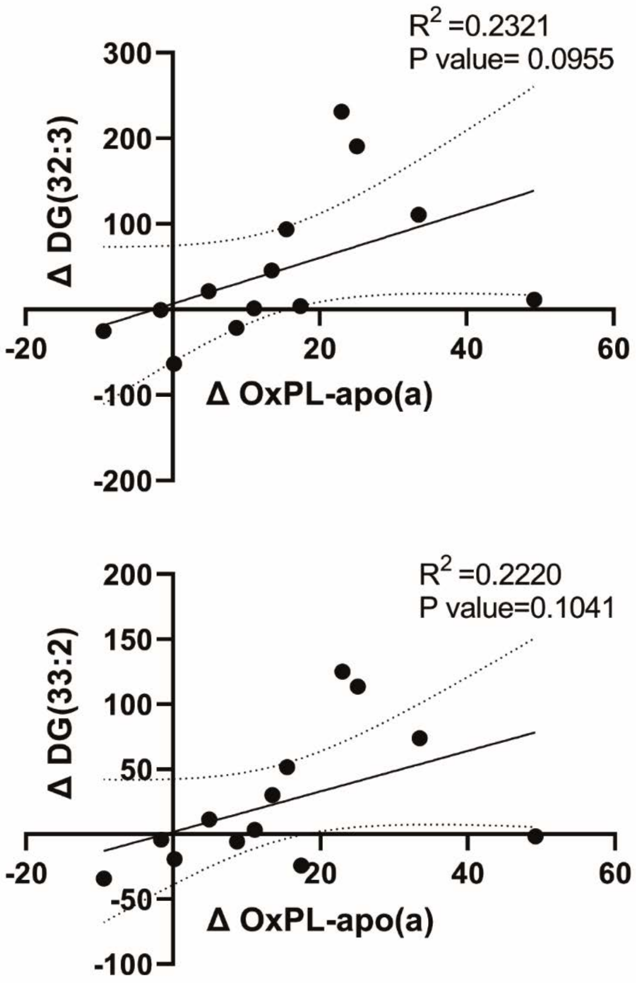

Figure S9: correlation plot between delta OxPL-apo(a) and delta DG(32:3) and delta DG(33:2).

Major resources table

Commercial lipids

Name	Vendor or source	Catalogue #	Lot#	Persistent ID/URL
Diacylglycerol 18:0-20:4 (1-stearoyl-2-arachidonoyl-sn-glycerol)	Avanti polar lipids (Alabama, USA)	800818C-10mg	800818C-10MG-A-018	https://avantilipids.com/product/800818
Diacylglycerol 18:0-22:6 (1-stearoyl-2-docosahexaenoyl-sn-glycerol)	Avanti polar lipids (Alabama, USA)	800819C-10mg	800819C-10MG-A-013	https://avantilipids.com/product/800819
Lysophosphatic acid 18:0 (1-stearoyl-2-hydroxy-sn-glycero-3-phosphate (sodium salt))	Avanti polar lipids (Alabama, USA)	857128P-25mg	857128P-25MG-A-042	https://avantilipids.com/product/857128

Antibodies

Target antigen	Vendor or source	Catalogue #	Working concentration	Lot#	Persistent ID/URL
NLRP3 (D4D8T)	Cell Signaling Technology	15101S	1:1000	4	https://www.cellsignal.com/products/primary-antibodies/nlrp3-d4d8t-rabbit-mab/15101
Caspase-1 (D7F10)	Cell Signaling Technology	3866T	1:1000	4	https://www.cellsignal.com/products/primary-antibodies/caspase-1-d7f10-rabbit-mab/3866
beta Actin (GT5512)	GeneTex	GTX629630	1:1000	41554	https://www.genetex.com/Product/Detail/beta-Actin-antibody-GT5512/GTX629630
Home Hsp90 antibody [AC88]	GeneTex	GTX13492	1:1000	822001926	https://www.genetex.com/Product/Detail/Hsp90-antibody-AC88/GTX13492

Peroxidase-Conjugated, Swine Anti-Rabbit Immunoglobulins	Dako	P0399	1:1000	20058714	http://www.finels.com/product/up_files/P0399.pdf
Peroxidase-Conjugated Rabbit Anti-Mouse Immunoglobulins	Dako	p0260	1:1000		http://www.finels.com/product/up_files/P0260.pdf
MARCO rabbit anti-human	Abcam	ab231046	1:500	GR-3244674-9)	
CD68 mouse anti-human; IgG3	Agilent	M0876	1:50	94759	
LPA4 mouse anti-human; IgG1	Kind gift from Prof. Sotirios Tsimikas EMD Millipore	#MABS1284	1:500		
GαRb IgG Alexa 488	Invitrogen	A11304	1:500	1616933	

GαMs IgG1 Alexa 568	Invitrogen	A21124	1:500	2129006	
GαMs IgG3 Alexa 647	Iackson ImmunoResearch	115-605-209	1:400	111911	
Hoechs	Thermo Fisher scientific	62249	1:500	KJ2279363	
E06 (mIgG)	Oxitope Pharma BV	OXI010101	300 ug/ml		

Cultured cells

Name	Vendor or source	Catalogue #	Lot#	Sex (F,M or unknown)	Persistent ID/URL

HAEC – Human Aortic Endothelial Cells	Lonza	CC-2535	#18TL036327		https://bioscience.lonza.com/lonza_bs/NL/en/Primary-and-Stem-Cells/p/000000000000184983/HAEC-%E2%80%93-Human-Aortic-Endothelial-Cells
