# Supplementary material for: Diacylglycerols and Lysophosphatidic Acid, Enriched on Lipoprotein(a), Contribute to Monocyte Inflammation
Source: Arterioscler Thromb Vasc Biol. 2024 Jan 25;44(3):720–40. doi: 10.1161/ATVBAHA.123.319937 (PMC10880937; doi:10.1161/ATVBAHA.123.319937)
Supplement: Supplementary file 10 [file atv-44-720-s010.pdf]

```
##=========================================================================================================
##=========================================================================================================
## LP(a) level analysis on macrophage populations
##=========================================================================================================
##=========================================================================================================
## Load data
dir.create("macrophage_subset_results/Jeff_LPa/", recursive = T, showWarnings = F)

# Lpa stratification
LPa.strat <- read.xlsx(file = "macrophage_subset_results/Jeff_LPa/scRNAseq AE with Lpa levels.xlsx", sheetIndex = 1)
LPa.strat

# Genes of interest
LPa.genes_of_interest.df <- read.xlsx(file = "macrophage_subset_results/Jeff_LPa/20230821_Genes of interest_ATVB rebuttal_single cell seq.xlsx", sheetIndex = 1, as.data.frame = T, header = T)
LPa.genes_of_interest.df

# Get rid of the NAs and put everything in to lists
LPa.genes_of_interest <- list()
for (i in colnames(LPa.genes_of_interest.df)){
  LPa.genes_of_interest[[i]] <- LPa.genes_of_interest.df[!is.na(LPa.genes_of_interest.df[,i]),i]
}
LPa.genes_of_interest

## Setup objects
# Add LPa metadata
all.seur.combined.M_clusters <- AddMetaData(all.seur.combined.M_clusters, metadata = merge(all.seur.combined.M_clusters@meta.data, LPa.strat, by.x = "Patient", by.y = "AE_Patient", all.x = T)$LPa, col.name = "LPa")
all.seur.combined.M_clusters <- AddMetaData(all.seur.combined.M_clusters, metadata = merge(all.seur.combined.M_clusters@meta.data, LPa.strat, by.x = "Patient", by.y = "AE_Patient", all.x = T)$LPa_rank, col.name = "LPa_rank")

# Define LP(a) rank colors
LPa.rank.colors       <- colorSpacer(startcolor = "cornflowerblue", endcolor = "chocolate", steps = 3, return.colors = T)
names(LPa.rank.colors) <- c("Low_10", "Mid", "High_10")

##=========================================================================================================
##=========================================================================================================
```

```
## Visualize LP(a) distribution
# UMAPs
p1 <- DimPlot(all.seur.combined.M_clusters, label = F, pt.size = 5,
group.by = "LPa_rank", cols = LPa.rank.colors, na.value =
"lightgrey")
p2 <- FeaturePlot(subset(all.seur.combined.M_clusters, LPa != "NA"),
features = "LPa", pt.size = 5, cols = c("cornflowerblue",
"chocolate"), order = T)
p3 <- DimPlot(all.seur.combined.M_clusters, label = F, pt.size = 5)
p1 + p2 + p3
ggsave("macrophage_subset_results/Jeff_LPa/
LPa_rank_cluster_combined_UMAP.pdf", width = 30, height = 10)

DimPlot(all.seur.combined.M_clusters, label = F, pt.size = 5,
group.by = "LPa_rank", cols = LPa.rank.colors, na.value =
"lightgrey")
ggsave("macrophage_subset_results/Jeff_LPa/LPa_rank_UMAP.pdf", width
= 15, height = 10)

FeaturePlot(all.seur.combined.M_clusters, features = "LPa", pt.size
= 5, cols = c("cornflowerblue", "chocolate"), shape.by = "LPa_rank",
order = T)
ggsave("macrophage_subset_results/Jeff_LPa/LPa_UMAP.pdf", width =
10, height = 10)

FeaturePlot(all.seur.combined.M_clusters, features = "LPa", pt.size
= 5, cols = c("cornflowerblue", "chocolate"), shape.by = "LPa_rank",
order = T, split.by = "ident")
ggsave("macrophage_subset_results/Jeff_LPa/
LPa_UMAP_ident_split.pdf", width = 50, height = 10, limitsize = F)

DimPlot(all.seur.combined.M_clusters, label = F, pt.size = 5)
ggsave("macrophage_subset_results/Jeff_LPa/cluster_UMAP.pdf", width
= 15, height = 10)

##==================================================================
=======================================
##==================================================================
=======================================
## LP(a) distribution over clusters
# Get LP(a) distribution per cluster
m.cluster.LPa.dist <- data.frame(row.names =
unique(Idents(all.seur.combined.M_clusters)))
for (theIdent in unique(Idents(all.seur.combined.M_clusters))){
  if(! is.null(ncol(subset(subset(all.seur.combined.M_clusters,
ident = theIdent), LPa_rank == "High_10", return.null = T)))){
    m.cluster.LPa.dist[theIdent, "High_10"] <-
ncol(subset(subset(all.seur.combined.M_clusters, ident = theIdent),
LPa_rank == "High_10", return.null = T))
  }else{
    m.cluster.LPa.dist[theIdent, "High_10"] <- 0
  }
  if(! is.null(ncol(subset(subset(all.seur.combined.M_clusters,
ident = theIdent), LPa_rank == "Low_10", return.null = T)))){
```

```
    m.cluster.LPa.dist[theIdent, "Low_10"]  <-
ncol(subset(subset(all.seur.combined.M_clusters, ident = theIdent),
LPa_rank == "Low_10",  return.null = T))
  }else{
    m.cluster.LPa.dist[theIdent, "Low_10"] <- 0
  }
  if(! is.null(ncol(subset(subset(all.seur.combined.M_clusters,
ident = theIdent), LPa_rank == "Mid", return.null = T)))){
    m.cluster.LPa.dist[theIdent, "Mid"] <-
ncol(subset(subset(all.seur.combined.M_clusters, ident = theIdent),
LPa_rank == "Mid",     return.null = T))
  }else{
    m.cluster.LPa.dist[theIdent, "Mid"] <- 0
  }
}
m.cluster.LPa.dist

# Add the total macrophage distribution
m.cluster.LPa.dist <- rbind(m.cluster.LPa.dist,
apply(m.cluster.LPa.dist, 2, sum))
row.names(m.cluster.LPa.dist)[4] <- "Total"
m.cluster.LPa.dist$Cluster <- row.names(m.cluster.LPa.dist)
m.cluster.LPa.dist

# Melt the frame
m <- melt(m.cluster.LPa.dist)
colnames(m) <- c("Cluster", "LPa", "Cells")
m$LPa <- factor(m$LPa, levels = c("Mid", "Low_10", "High_10"))
m

# Plot distribution
ggplot(m, aes(x = Cluster, y = Cells, fill = LPa)) +
  geom_bar(stat = "identity", position = "fill", width = 0.9) +
  scale_fill_manual(values = LPa.rank.colors) +
  ylab("% of Cells per cluster") + theme_pubr() + theme(aspect.ratio
= 3, axis.text.x = element_text(angle =45, hjust = 1))
ggsave("macrophage_subset_results/Jeff_LPa/LP(a) distribution over
clusters.pdf")

# Calculate ratios
high_over_low.LPa_ratio        <- m.cluster.LPa.dist$High_10 /
m.cluster.LPa.dist$Low_10
names(high_over_low.LPa_ratio) <- m.cluster.LPa.dist$Cluster
scaled.high_over_low.LPa_ratio <- log2(high_over_low.LPa_ratio)

# Melt it
m <- data.frame("Scaled_Ratio" = scaled.high_over_low.LPa_ratio,
row.names = names(scaled.high_over_low.LPa_ratio))
m$Cluster <- row.names(m)
m
# Remove infinite row
m <- m[is.finite(m[,"Scaled_Ratio"]),]

# And plot the scaled ratios
```

```
ggplot(m, aes(x = Cluster, y = Scaled_Ratio, fill = Scaled_Ratio)) +
  geom_bar(stat = "identity") +
  scale_fill_gradient(low = LPa.rank.colors["Low_10"], high =
LPa.rank.colors["High_10"]) +
  ylab("Log2(High_10 # Cells / Low_10 # Cells)")  + theme_pubr() +
theme(aspect.ratio = 2, axis.text.x = element_text(angle =45, hjust
= 1))
ggsave("macrophage_subset_results/Jeff_LPa/LP(a) distribution ratio
over clusters.pdf")

##==================================================================
=======================================
##==================================================================
=======================================
## Genes of interest in LP(a) groups
dir.create("macrophage_subset_results/Jeff_LPa/genes_of_interest/
per_cluster", recursive = T, showWarnings = F)
dir.create("macrophage_subset_results/Jeff_LPa/genes_of_interest/
per_LP(a)_rank", recursive = T, showWarnings = F)
dir.create("macrophage_subset_results/Jeff_LPa/genes_of_interest/
per_cluster_and_LP(a)_rank", recursive = T, showWarnings = F)

# Per cluster
for (i in names(LPa.genes_of_interest)){
  DoHeatmap(all.seur.combined.M_clusters, features =
LPa.genes_of_interest[[i]], raster = F)
  ggsave(paste("macrophage_subset_results/Jeff_LPa/
genes_of_interest/per_cluster/Genes of interest - per cluster - ",
i, " - heatmap.pdf", sep = ""))

  VlnPlot(all.seur.combined.M_clusters, features =
LPa.genes_of_interest[[i]], ncol = 4)
  ggsave(paste("macrophage_subset_results/Jeff_LPa/
genes_of_interest/per_cluster/Genes of interest - per cluster - ",
i, " - violinplot.pdf", sep = ""))

  DotPlot(all.seur.combined.M_clusters, features =
LPa.genes_of_interest[[i]], cols = c("cornflowerblue", "chocolate"),
dot.scale = 15)
  ggsave(paste("macrophage_subset_results/Jeff_LPa/
genes_of_interest/per_cluster/Genes of interest - per cluster - ",
i, " - dotplot.pdf", sep = ""), width = 17)

  FeaturePlot(all.seur.combined.M_clusters, features =
LPa.genes_of_interest[[i]], pt.size = 2, cols = c("cornflowerblue",
"chocolate"), order = T, ncol = 4)
  ggsave(paste("macrophage_subset_results/Jeff_LPa/
genes_of_interest/per_cluster/Genes of interest - per cluster - ",
i, " - featureplot.pdf", sep = ""), width = 15, height = 15)
}

# Per LP(a) strat
for (i in names(LPa.genes_of_interest)){
  DoHeatmap(subset(all.seur.combined.M_clusters, LPa_rank != "NA"),
```

```
features = LPa.genes_of_interest[[i]], group.by = "LPa_rank", raster
= F, group.colors = LPa.rank.colors)
  ggsave(paste("macrophage_subset_results/Jeff_LPa/
genes_of_interest/per_LP(a)_rank/Genes of interest - per LP(a) rank
- ", i, " - heatmap.pdf", sep = ""))

  VlnPlot(subset(all.seur.combined.M_clusters, LPa_rank != "NA"),
features = LPa.genes_of_interest[[i]], group.by = "LPa_rank", ncol =
4, cols = LPa.rank.colors)
  ggsave(paste("macrophage_subset_results/Jeff_LPa/
genes_of_interest/per_LP(a)_rank/Genes of interest - per LP(a) rank
- ", i, " - violinplot.pdf", sep = ""))

  DotPlot(subset(all.seur.combined.M_clusters, LPa_rank != "NA"),
features = LPa.genes_of_interest[[i]], group.by = "LPa_rank", cols =
c("cornflowerblue", "chocolate"), dot.scale = 15) +
theme(aspect.ratio = 0.3)
  ggsave(paste("macrophage_subset_results/Jeff_LPa/
genes_of_interest/per_LP(a)_rank/Genes of interest - per LP(a) rank
- ", i, " - dotplot.pdf", sep = ""), width = 17, height = 5)

  FeaturePlot(subset(all.seur.combined.M_clusters, LPa_rank !=
"NA"), features = LPa.genes_of_interest[[i]], shape.by = "LPa_rank",
pt.size = 2, cols = c("cornflowerblue", "chocolate"), order = T,
ncol = 4)
  ggsave(paste("macrophage_subset_results/Jeff_LPa/
genes_of_interest/per_LP(a)_rank/Genes of interest - per LP(a) rank
- ", i, " - featureplot.pdf", sep = ""), width = 25, height = 25)
}

# Per cluster and LP(a) strat
# Heatmaps split per cluster
for (i in names(LPa.genes_of_interest)){
  for (theIdent in unique(Idents(all.seur.combined.M_clusters))){
    DoHeatmap(subset(subset(all.seur.combined.M_clusters, ident =
theIdent), LPa_rank != "NA"), features = LPa.genes_of_interest[[i]],
raster = F, group.by = "LPa_rank", group.colors = LPa.rank.colors)
    ggsave(paste("macrophage_subset_results/Jeff_LPa/
genes_of_interest/per_cluster_and_LP(a)_rank/Genes of interest -
cluster ", theIdent, " - per LP(a) rank - ", i, " - heatmap.pdf",
sep = ""))
  }
}

# Violins and dot plots
for (i in names(LPa.genes_of_interest)){
  # Careful, VLnPlot split.by doesn't honor the names of the colors
passed to it AND doesn't show the legend if more than one feature is
plotted! So need to to adjust manually and check on a single gene or
make an explicit call to legend.position in theme.
  VlnPlot(subset(all.seur.combined.M_clusters, LPa_rank != "NA"),
features = LPa.genes_of_interest[[i]], split.by = "LPa_rank", ncol =
4, cols = LPa.rank.colors[c(3,1,2)]) & theme(aspect.ratio = 0.5)
  ggsave(paste("macrophage_subset_results/Jeff_LPa/
```

```
genes_of_interest/per_cluster_and_LP(a)_rank/Genes of interest - all
clusters - per LP(a) rank - ", i, " - violinplot.pdf", sep = ""),
width = 20, height = 15)

  DotPlot(subset(all.seur.combined.M_clusters, LPa_rank != "NA"),
features = LPa.genes_of_interest[[i]], split.by = "LPa_rank", cols =
LPa.rank.colors, dot.scale = 15) + theme(aspect.ratio = 0.75)
  ggsave(paste("macrophage_subset_results/Jeff_LPa/
genes_of_interest/per_cluster_and_LP(a)_rank/Genes of interest - all
clusters - per LP(a) rank - ", i, " - dotplot.pdf", sep = ""), width
= 17, height = 12)
}


##=================================================================
=======================================
##=================================================================
=======================================
## Differential expression of genes within the LP(a) groups
dir.create("macrophage_subset_results/Jeff_LPa/marker_genes/
LPa_rank_DE", recursive = T, showWarnings = F)

# Define markers globally
LPa.high_low.markers <-
FindMarkers(subset(all.seur.combined.M_clusters, LPa_rank != "NA"),
group.by = "LPa_rank", ident.1 = "High_10", ident.2 = "Low_10",
logfc.threshold = 0.25, min.pct = 0.25)
head(LPa.high_low.markers)

LPa.high_all.markers <-
FindMarkers(subset(all.seur.combined.M_clusters, LPa_rank != "NA"),
group.by = "LPa_rank", ident.1 = "High_10", logfc.threshold = 0.25,
min.pct = 0.25)
head(LPa.high_all.markers)

LPa.low_all.markers <-
FindMarkers(subset(all.seur.combined.M_clusters, LPa_rank != "NA"),
group.by = "LPa_rank", ident.1 = "Low_10", logfc.threshold = 0.25,
min.pct = 0.25)
head(LPa.low_all.markers)

# Plot the sole sig marker...
VlnPlot(subset(all.seur.combined.M_clusters, LPa_rank != "NA"),
features = row.names(LPa.high_all.markers)[1], cols =
LPa.rank.colors[c(3,1,2)], group.by = "LPa_rank")
ggsave(paste("macrophage_subset_results/Jeff_LPa/marker_genes/
LPa_rank_DE/High_10 vs All sig DE gene - ",
row.names(LPa.high_all.markers)[1], " - violinplot.pdf"))

##=================================================================
=======================================
##=================================================================
=======================================
## Differential expression of genes within the LP(a) groups per
```

```
cluster
dir.create("macrophage_subset_results/Jeff_LPa/marker_genes/
per_cluster_LPa_rank_DE", recursive = T, showWarnings = F)

# Setup vars
LPa.clusters.high_low.markers <- list()
LPa.clusters.high_all.markers <- list()
LPa.clusters.low_all.markers  <- list()

# Define markers per cluster
# Check if there are more than two cells in each subset or the
funciton throws an error
for (theIdent in unique(Idents(all.seur.combined.M_clusters))){
  if(ncol(subset(subset(all.seur.combined.M_clusters, ident =
theIdent), LPa_rank == "Low_10")) > 2 &
ncol(subset(subset(all.seur.combined.M_clusters, ident = theIdent),
LPa_rank == "High_10")) > 2){
    LPa.clusters.high_low.markers[[theIdent]] <-
FindMarkers(subset(subset(all.seur.combined.M_clusters, ident =
theIdent), LPa_rank != "NA"), group.by = "LPa_rank", ident.1 =
"High_10", ident.2 = "Low_10", logfc.threshold = 0.25, min.pct =
0.25)
  }
  if(ncol(subset(subset(all.seur.combined.M_clusters, ident =
theIdent), LPa_rank == "High_10")) > 2){
    LPa.clusters.high_all.markers[[theIdent]] <-
FindMarkers(subset(subset(all.seur.combined.M_clusters, ident =
theIdent), LPa_rank != "NA"), group.by = "LPa_rank", ident.1 =
"High_10", logfc.threshold = 0.25, min.pct = 0.25)
  }
  if(ncol(subset(subset(all.seur.combined.M_clusters, ident =
theIdent), LPa_rank == "Low_10")) > 2){
    LPa.clusters.low_all.markers[[theIdent]]  <-
FindMarkers(subset(subset(all.seur.combined.M_clusters, ident =
theIdent), LPa_rank != "NA"), group.by = "LPa_rank", ident.1 =
"Low_10",  logfc.threshold = 0.25, min.pct = 0.25)
  }
}

# Check number sig DE genes
lapply(LPa.clusters.high_low.markers, function(x)sum(x[,"p_val_adj"]
< 0.05))
lapply(LPa.clusters.high_all.markers, function(x)sum(x[,"p_val_adj"]
< 0.05))
lapply(LPa.clusters.low_all.markers,  function(x)sum(x[,"p_val_adj"]
< 0.05))

# Head the sig hits
head(LPa.clusters.high_all.markers$`CD68+ABCA1+OLR1+TREM2+ Foam
Cells`)
head(LPa.clusters.high_low.markers$`CD68+ABCA1+OLR1+TREM2+ Foam
Cells`)

# And let's plot them...
```

```
VlnPlot(subset(all.seur.combined.M_clusters, LPa_rank != "NA"),
features =
c(row.names(LPa.clusters.high_all.markers$`CD68+ABCA1+OLR1+TREM2+
Foam Cells`)
[1:2],row.names(LPa.clusters.high_low.markers$`CD68+ABCA1+OLR1+TREM2
+ Foam Cells`)[1]), cols = LPa.rank.colors[c(3,1,2)], split.by =
"LPa_rank")
ggsave(paste("macrophage_subset_results/Jeff_LPa/marker_genes/
per_cluster_LPa_rank_DE/High_10 vs All cluster 3 - sig DE gene - ",
row.names(LPa.clusters.high_all.markers$`3`)[1], " -
violinplot.pdf"))


##==================================================================
========================================
##==================================================================
========================================
## Now let's soleley look at the low and high LP(a) groups
##==================================================================
========================================
##==================================================================
========================================
dir.create("macrophage_subset_results/Jeff_LPa/high_low_only/",
showWarnings = F, recursive = T)

## LP(a) distribution over clusters
# Get LP(a) distribution per cluster
m.cluster.LPa.dist <- data.frame(row.names =
unique(Idents(all.seur.combined.M_clusters)))
for (theIdent in unique(Idents(all.seur.combined.M_clusters))){
  if(! is.null(ncol(subset(subset(all.seur.combined.M_clusters,
ident = theIdent), LPa_rank == "High_10", return.null = T)))){
    m.cluster.LPa.dist[theIdent, "High_10"] <-
ncol(subset(subset(all.seur.combined.M_clusters, ident = theIdent),
LPa_rank == "High_10", return.null = T))
  }else{
    m.cluster.LPa.dist[theIdent, "High_10"] <- 0
  }
  if(! is.null(ncol(subset(subset(all.seur.combined.M_clusters,
ident = theIdent), LPa_rank == "Low_10", return.null = T)))){
    m.cluster.LPa.dist[theIdent, "Low_10"]  <-
ncol(subset(subset(all.seur.combined.M_clusters, ident = theIdent),
LPa_rank == "Low_10",  return.null = T))
  }else{
    m.cluster.LPa.dist[theIdent, "Low_10"] <- 0
  }
}
m.cluster.LPa.dist

# Add the total macrophage distribution
m.cluster.LPa.dist <- rbind(m.cluster.LPa.dist,
apply(m.cluster.LPa.dist, 2, sum))
row.names(m.cluster.LPa.dist)[4] <- "Total"
m.cluster.LPa.dist$Cluster <- row.names(m.cluster.LPa.dist)
```

```
m.cluster.LPa.dist

# Melt the frame
m <- melt(m.cluster.LPa.dist)
colnames(m) <- c("Cluster", "LPa", "Cells")
m$LPa <- factor(m$LPa, levels = c("Low_10", "High_10"))
m

# Plot distribution
ggplot(m, aes(x = Cluster, y = Cells, fill = LPa)) +
  geom_bar(stat = "identity", position = "fill", width = 0.9) +
  scale_fill_manual(values = LPa.rank.colors[c(1,3)]) +
  ylab("% of Cells per cluster") + theme_pubr() + theme(aspect.ratio
= 3, axis.text.x = element_text(angle =45, hjust = 1))
ggsave("macrophage_subset_results/Jeff_LPa/high_low_only/LP(a)
distribution over clusters.pdf")

# Calculate ratios
high_over_low.LPa_ratio        <- m.cluster.LPa.dist$High_10 /
m.cluster.LPa.dist$Low_10
names(high_over_low.LPa_ratio) <- m.cluster.LPa.dist$Cluster
scaled.high_over_low.LPa_ratio <- log2(high_over_low.LPa_ratio)

# Melt it
m <- data.frame("Scaled_Ratio" = scaled.high_over_low.LPa_ratio,
row.names = names(scaled.high_over_low.LPa_ratio))
m$Cluster <- row.names(m)
m
# Remove infinite row
m <- m[is.finite(m[,"Scaled_Ratio"]),]

# And plot the scaled ratios
ggplot(m, aes(x = Cluster, y = Scaled_Ratio, fill = Scaled_Ratio)) +
  geom_bar(stat = "identity") +
  scale_fill_gradient(low = LPa.rank.colors["Low_10"], high =
LPa.rank.colors["High_10"]) +
  ylab("Log2(High_10 # Cells / Low_10 # Cells)")  + theme_pubr() +
theme(aspect.ratio = 2, axis.text.x = element_text(angle =45, hjust
= 1))
ggsave("macrophage_subset_results/Jeff_LPa/high_low_only/LP(a)
distribution ratio over clusters.pdf")

##==================================================================
=========================================
##==================================================================
=========================================
## Genes of interest in LP(a) groups
dir.create("macrophage_subset_results/Jeff_LPa/high_low_only/
genes_of_interest/per_cluster", recursive = T, showWarnings = F)
dir.create("macrophage_subset_results/Jeff_LPa/high_low_only/
genes_of_interest/per_LP(a)_rank", recursive = T, showWarnings = F)
dir.create("macrophage_subset_results/Jeff_LPa/high_low_only/
genes_of_interest/per_cluster_and_LP(a)_rank", recursive = T,
showWarnings = F)
```

```
# Per cluster
for (i in names(LPa.genes_of_interest)){
  DoHeatmap(all.seur.combined.M_clusters, features =
LPa.genes_of_interest[[i]], raster = F)
  ggsave(paste("macrophage_subset_results/Jeff_LPa/high_low_only/
genes_of_interest/per_cluster/Genes of interest - per cluster - ",
i, " - heatmap.pdf", sep = ""))

  VlnPlot(all.seur.combined.M_clusters, features =
LPa.genes_of_interest[[i]], ncol = 4)
  ggsave(paste("macrophage_subset_results/Jeff_LPa/high_low_only/
genes_of_interest/per_cluster/Genes of interest - per cluster - ",
i, " - violinplot.pdf", sep = ""))

  DotPlot(all.seur.combined.M_clusters, features =
LPa.genes_of_interest[[i]], cols = c("cornflowerblue", "chocolate"),
dot.scale = 15)
  ggsave(paste("macrophage_subset_results/Jeff_LPa/high_low_only/
genes_of_interest/per_cluster/Genes of interest - per cluster - ",
i, " - dotplot.pdf", sep = ""), width = 17)

  FeaturePlot(all.seur.combined.M_clusters, features =
LPa.genes_of_interest[[i]], pt.size = 2, cols = c("cornflowerblue",
"chocolate"), order = T, ncol = 4)
  ggsave(paste("macrophage_subset_results/Jeff_LPa/high_low_only/
genes_of_interest/per_cluster/Genes of interest - per cluster - ",
i, " - featureplot.pdf", sep = ""), width = 15, height = 15)
}

# Per LP(a) strat
for (i in names(LPa.genes_of_interest)){
  DoHeatmap(subset(all.seur.combined.M_clusters, LPa_rank != "NA" &
LPa_rank != "Mid"),   features = LPa.genes_of_interest[[i]],
group.by = "LPa_rank", raster = F, group.colors =
LPa.rank.colors[c(1,3)])
  ggsave(paste("macrophage_subset_results/Jeff_LPa/high_low_only/
genes_of_interest/per_LP(a)_rank/Genes of interest - per LP(a) rank
- ", i, " - heatmap.pdf", sep = ""))

  VlnPlot(subset(all.seur.combined.M_clusters, LPa_rank != "NA" &
LPa_rank != "Mid"),     features = LPa.genes_of_interest[[i]],
group.by = "LPa_rank", ncol = 4, cols = LPa.rank.colors[c(1,3)])
  ggsave(paste("macrophage_subset_results/Jeff_LPa/high_low_only/
genes_of_interest/per_LP(a)_rank/Genes of interest - per LP(a) rank
- ", i, " - violinplot.pdf", sep = ""))

  DotPlot(subset(all.seur.combined.M_clusters, LPa_rank != "NA" &
LPa_rank != "Mid"),     features = LPa.genes_of_interest[[i]],
group.by = "LPa_rank", cols = c("cornflowerblue", "chocolate"),
dot.scale = 15) + theme(aspect.ratio = 0.3)
  ggsave(paste("macrophage_subset_results/Jeff_LPa/high_low_only/
genes_of_interest/per_LP(a)_rank/Genes of interest - per LP(a) rank
- ", i, " - dotplot.pdf", sep = ""), width = 17, height = 5)
```

```
  FeaturePlot(subset(all.seur.combined.M_clusters, LPa_rank != "NA"
& LPa_rank != "Mid"), features = LPa.genes_of_interest[[i]],
shape.by = "LPa_rank", pt.size = 2, cols = c("cornflowerblue",
"chocolate"), order = T, ncol = 4)
  ggsave(paste("macrophage_subset_results/Jeff_LPa/high_low_only/
genes_of_interest/per_LP(a)_rank/Genes of interest - per LP(a) rank
- ", i, " - featureplot.pdf", sep = ""), width = 25, height = 25)
}

# Per cluster and LP(a) strat
# Heatmaps split per cluster
for (i in names(LPa.genes_of_interest)){
  for (theIdent in unique(Idents(all.seur.combined.M_clusters))){
    DoHeatmap(subset(subset(all.seur.combined.M_clusters, ident =
theIdent), LPa_rank != "NA"), features = LPa.genes_of_interest[[i]],
raster = F, group.by = "LPa_rank", group.colors =
LPa.rank.colors[c(1,3)])
    ggsave(paste("macrophage_subset_results/Jeff_LPa/high_low_only/
genes_of_interest/per_cluster_and_LP(a)_rank/Genes of interest -
cluster ", theIdent, " - per LP(a) rank - ", i, " - heatmap.pdf",
sep = ""))
  }
}

# Violins and dot plots
for (i in names(LPa.genes_of_interest)){
  # Careful, VLnPlot split.by doesn't honor the names of the colors
passed to it AND doesn't show the legend if more than one feature is
plotted! So need to to adjust manually and check on a single gene or
make an explicit call to legend.position in theme.
  VlnPlot(subset(all.seur.combined.M_clusters, LPa_rank != "NA" &
LPa_rank != "Mid"), features = LPa.genes_of_interest[[i]], split.by
= "LPa_rank", ncol = 4, cols = LPa.rank.colors[c(3,1)]) &
theme(aspect.ratio = 0.5)
  ggsave(paste("macrophage_subset_results/Jeff_LPa/high_low_only/
genes_of_interest/per_cluster_and_LP(a)_rank/Genes of interest - all
clusters - per LP(a) rank - ", i, " - violinplot.pdf", sep = ""),
width = 20, height = 15)

  DotPlot(subset(all.seur.combined.M_clusters, LPa_rank != "NA" &
LPa_rank != "Mid"), features = LPa.genes_of_interest[[i]], split.by
= "LPa_rank", cols = LPa.rank.colors[c(3,1,2)], dot.scale = 15) +
theme(aspect.ratio = 0.75)
  ggsave(paste("macrophage_subset_results/Jeff_LPa/high_low_only/
genes_of_interest/per_cluster_and_LP(a)_rank/Genes of interest - all
clusters - per LP(a) rank - ", i, " - dotplot.pdf", sep = ""), width
= 17, height = 12)
}


##============================================================
=======================================
##============================================================
```

```
=======================================
## Differential expression of genes within the LP(a) groups
dir.create("macrophage_subset_results/Jeff_LPa/high_low_only/
marker_genes/LPa_rank_DE", recursive = T, showWarnings = F)

# Define markers globally
LPa.high_low.markers <-
FindMarkers(subset(all.seur.combined.M_clusters, LPa_rank != "NA" &
LPa_rank != "Mid"), group.by = "LPa_rank", ident.1 = "High_10",
ident.2 = "Low_10", logfc.threshold = 0.25, min.pct = 0.25)
head(LPa.high_low.markers)


##==================================================================
=======================================
##==================================================================
=======================================
## Differential expression of genes within the LP(a) groups per
cluster
dir.create("macrophage_subset_results/Jeff_LPa/high_low_only/
marker_genes/per_cluster_LPa_rank_DE", recursive = T, showWarnings =
F)

# Setup vars
LPa.clusters.high_low.markers <- list()

# Define markers per cluster
# Check if there are more than two cells in each subset or the
funciton throws an error
for (theIdent in unique(Idents(all.seur.combined.M_clusters))){
  if(ncol(subset(subset(all.seur.combined.M_clusters, ident =
theIdent), LPa_rank == "Low_10")) > 2 &
ncol(subset(subset(all.seur.combined.M_clusters, ident = theIdent),
LPa_rank == "High_10")) > 2){
    LPa.clusters.high_low.markers[[theIdent]] <-
FindMarkers(subset(subset(all.seur.combined.M_clusters, ident =
theIdent), LPa_rank != "NA"), group.by = "LPa_rank", ident.1 =
"High_10", ident.2 = "Low_10", logfc.threshold = 0.25, min.pct =
0.25)
  }
}

# Check number sig DE genes
lapply(LPa.clusters.high_low.markers, function(x)sum(x[,"p_val_adj"]
< 0.05))
head(LPa.clusters.high_low.markers$`CD68+ABCA1+OLR1+TREM2+ Foam
Cells`)
```
